# A deep learning based steganography integration framework for ad-hoc cloud computing data security augmentation using the V-BOINC system

**DOI:** 10.1186/s13677-022-00339-w

**Published:** 2022-12-21

**Authors:** Ahmed A. Mawgoud, Mohamed Hamed N. Taha, Amr Abu-Talleb, Amira Kotb

**Affiliations:** 1 Information Technology Department, Faculty of Computers and Artificial Intelligence, Cairo University, Giza, Egypt; 2 Mechatronics Department, Faculty of Engineering, German University in Cairo, Cairo, Egypt

**Keywords:** Ad-hoc system, Cloud computing, Steganography, Cloud security, Deep learning, Encryption

## Abstract

In the early days of digital transformation, the automation, scalability, and availability of cloud computing made a big difference for business. Nonetheless, significant concerns have been raised regarding the security and privacy levels that cloud systems can provide, as enterprises have accelerated their cloud migration journeys in an effort to provide a remote working environment for their employees, primarily in light of the COVID-19 outbreak. The goal of this study is to come up with a way to improve steganography in ad hoc cloud systems by using deep learning. This research implementation is separated into two sections. In Phase 1, the “Ad-hoc Cloud System” idea and deployment plan were set up with the help of V-BOINC. In Phase 2, a modified form of steganography and deep learning were used to study the security of data transmission in ad-hoc cloud networks. In the majority of prior studies, attempts to employ deep learning models to augment or replace data-hiding systems did not achieve a high success rate. The implemented model inserts data images through colored images in the developed ad hoc cloud system. A systematic steganography model conceals from statistics lower message detection rates. Additionally, it may be necessary to incorporate small images beneath huge cover images. The implemented ad-hoc system outperformed Amazon AC2 in terms of performance, while the execution of the proposed deep steganography approach gave a high rate of evaluation for concealing both data and images when evaluated against several attacks in an ad-hoc cloud system environment.

## Introduction

During the last decade, the cloud computing became one of the massive growing technologies, it provided both automated and orchestrated solution for both individuals and corporates; its importance appeared in the acceleration of the digital transformation processes worldwide during the era of COVID-19. Recently, cybersecurity threats have risen at a high rate [1]. Cloud security is always the big topic when it comes to any sort of edge computing process. Many research topics provide various techniques for enhancing cloud security. However, there is a huge room for contributions regarding the critical security concerns regarding cloud systems [2]. The ability to utilize existing resources to supply cloud services within a scope of unreliable hosts in comparison with one provided by 'Grid Computing'. Instead, the ‘Ad-hoc Cloud Model’ concept may be somehow comparable to the ‘Volunteer Computing’, the paradigm itself of the ad-hoc cloud systems including various additional keys [3].

### The ad-hoc cloud computing paradigm

The design of the (Ad-hoc Cloud Computing) is having a high rate of similarity of the classic (Grid Computing), (Condor) [4] and (Volunteer Computing System) based on Berkeley Open Infrastructure of Network Computing. As the main idea is to re-use the host computing accessible resources for operational tasks. Nevertheless, the chosen computational design for resources utilization has various encounters, which should be highlight to provide a high performance evaluation for the end-user [5]. Consequently, multiple standards should be implemented to provide the same features of the ordinary cloud computing system. However, there were multiple key differences the unified the ad-hoc computing system design:

- The system can work within a group of periodically accessible hosts, which might possibly have some un- excepted behavior from time to another [6].
- The provided resources from a cloud cluster or a grid node can be dedicated to serve a single ad-hoc cloud node.
- The end-user implies a consistent level of trust towards the volunteer resources and grid systems; as there is no existence for a trust relation between the infrastructure system and the end user.
- Business continuity can be provided through a group of unreliable nodes, the ad-hoc cloud system provides the availability for both host and guest users within the ad-hoc cloud system in case any failure case occurred periodically [7].
- The operated host processes –regardless the resources’ consumption level- were not affected by any means with the ad-hoc cloud system over the passing time.
- The volunteer system includes various wide range of options as resources (i.e. Disk Space, Memory and I\O) [8].

### Deep steganography

Steganography is the art of hiding data or images within another image; the term was coined in the 15th century, when communications were physically hidden [9]. Currently, steganography is a form of encryption technique. Steganography offers a challenge since it can alter the appearance and content of the carrier. Two factors affect the degree of variation: Initially, the magnitude of material was suppressed. Images have traditionally been used to disguise messages within written text. The information is concealed behind an image [10]. Bits-per-pixel (bpp) is the unit of measurement for the hidden data rate. Most of the time, this method limits the total amount of data to 0.3 bpp or less. As the message length increases, so does the bpp, and the amount of change depends on the resolution of the original image [11].

### Motivation

As a result of the high complexity of cloud infrastructure operations, such as [12–14], as well as the existence of unreliable resources, there were numerous obstacles that needed to be addressed. These types of difficulties and the essential techniques for overcoming them were discussed in great depth. It is challenging to develop a cloud solution prototype with a high-level data security paradigm. The performance of LAN security may differ from that of other network types [15]. It is well-known that steganography can be used to conceal data for a variety of purposes, including to perform malicious acts using graphics on websites that conceal data [16]. Digital watermarks, on the other hand, could be used to add data or images without degrading the image quality. As embedding a message alters the appearance and essential properties of the carrier [17], previously proposed successful steganography systems have experienced significant challenges. The most common impediments consist of two points:

- The amount of the required data to be
- The level of change that must be achieved by the used

It is also essential to note that the extent of change depends on the image itself. Utilizing high-frequency image parts to conceal data resulted in fewer perceptible interruptions [18] compared to using low-frequency image sections. Various common steganography techniques use the images' 'Least Significant Bits' (LSB) for secret data hiding, if it is completed with flexibility and consistency, as it is statistically difficult to observe the output files' alteration rate for multimedia data (i.e., images, audio, and video) [19]. Techniques such as HUGO, which construct and match possible varieties of 'Cover Image' clones based upon their first order attributes, strive to maintain image statistics if afflicted images differ from their unaffected counterparts. HUGO is often used for communications with a size of less than 0.5 bpp [20]. So, neural networks were mostly used to explicitly predict the availability of natural visuals and to embed the whole photos in carrier graphics in a much more efficient way than in previous studies [21].

### Study contributions

This main target of this study was divided into two phases, with the purpose of introducing a full approach to:

A. Implement an ad-hoc cloud integrated architecture as an end-to-end solution and then evaluate both performance and privacy standards within the implemented technique:
  i. Design a steganography model for securing data and images transmission in the ad-hoc cloud system through using deep learning.
  ii. Develop an ad-hoc cloud framework through absorbing services through irregular and unstable networks.

This study assumes that the technology should be used primarily on LAN networks as objectives were aiming for both availability and efficiency. WAN networks can be utilized for evaluating the security measurements. Systems that demand exceptional security might not be efficient for the ad-hoc cloud architecture. Whenever a host crashes unexpectedly, data discrepancies can cause problems for applications, including those who communicate to external monitoring.

B. Design a steganography technique structure with deep learning utilization:
  i. The implemented model should be able to conceal a ‘Secret Image’ with overall N × N × RGB pixels throughout a ‘Cover Image’ with its original form ‘color channel = 8 bits’.
  ii. Having the ability to provide a successful approach for reducing the constraints through which the ‘Secret Image’ has delivered without any loss of the image quality, apart from those researches.
  iii. The main challenge here is to compromise both reliability and the carrier security along with the ‘Secret Image’.

### Paper organization

The organization of the paper is as following: Section 2 highlights the both concepts of ad-hoc cloud systems and steganography into ad-hoc cloud model. Section 3 describes the challenges that motivates the author for this study. Section 4 has introduced the proposed ad-hoc cloud system model and an illustration of the approach of utilizing deep learning networks with steganography. Section 5, an illustration of the ad-hoc cloud system implementation model and the training network approach for steganography. Section 6 has defined a discussion regarding the output result and the experiment performance measurements, then an illustration of the output limitations. Section 7 highlights both discussion and analysis regarding the output of both strengths and weaknesses. Section 8, it has provided a summarization regarding the overall study work.

## Literature review

Recently, the definition of ad-hoc cloud computing as a definition was identified to explore various areas as a part of the transformation phase from classic LAN/WAN wireless networks into cloud systems; in order to implement a distributed infrastructure - using non-exclusive and intermittently accessible hosts and devices [22]. There were two major existing projects that were discussed for both ad-hoc cloud systems and volunteer computing, and the present state of research, Table 1 below states the description of the mentioned acronyms in the whole paper.

**Table 1:** Abbreviations Description

Acronym	Description
API	Application Programming Interface
ER	Bit Error Rate
Bpp	Bits per pixel
BOINC	Berkeley Open Infrastructure for Network Computing
CA	Cloud Jobs Assigned
CC	Cloud Jobs Completed
CDTF	Camera Display Transfer Function
CNN	Convolutional Neural Networks
DB	Database
DCGAN	Deep Convolutional Generative Adversarial Networks
DCT	Discrete Cosine Transform
DDH	Decisional Diffie–Hellman
DNN	Deep Neural Networks
ECC	Elliptic Curve Cryptography
Gmond	Ganglia Monitoring Daemon
GUI	Graphical User Interface
gUse	Grid and User Support Environment
HUGO	Highly Undetectable steGO
LAN	Local Area Network
LSB	Least Significant Bit
LFM	linear frequency modulation
MSB	Most Significant Bits
NCC	Normalized Cross Correlation
NF	Number of Failures
OS	Operating System
P2P	Peer to Peer
QoS	Quality of Service
RAM	Random Access Memory
RGB	Red Green Blue
RRD	Redundancy Rate Distortion
ROC	Report on Compliance
SLA	Service Level Agreement
SOAP	Simple Object Access Protocol
SSE	Secret Space Encryptor
SSIM	Structural Similarity Index Measure
V-BOINC	Virtualized Berkeley Open Infrastructure for Network Computing
VDI	Virtual Desktop Infrastructure
VM	Virtual Machine
WAN	Wide Area Network
WS-PGRADE	Web Services - Parallel Grid Runtime and Developer Environment
UDH	Unsigned Diffie-Hellman
XML	Extensible Markup Language

The idea of converting the virtualized infrastructure system into an ad-hoc cloud system have been discussed in this section, as we present:

- A discussion about previous proposed studies regarding the ad-hoc cloud systems, this was done through highlighting two key publications that explain the definition as well as providing extended ideas for future study.
- The related research aiming to turn volunteer computing to have the features of cloud systems was addressed.
- The ad-hoc cloud prototype’s framework, execution and approach illustration from which the distributed volunteer infrastructure turns into a centralized infrastructure.

Through the utilization of the ad-hoc cloud system, the user has the ability submit a work to BOINC with the utilization of a modified V-BOINC server, then the VM may be scheduled to the nearest optimum ad-hoc host [4]. The essential purpose for implemented cloud system is to bring reliability to the un-stable infrastructure V-BOINC, regular check pointing, rescheduling, and recovery were discussed in details [23].

### Related work

**Fig. 1:**
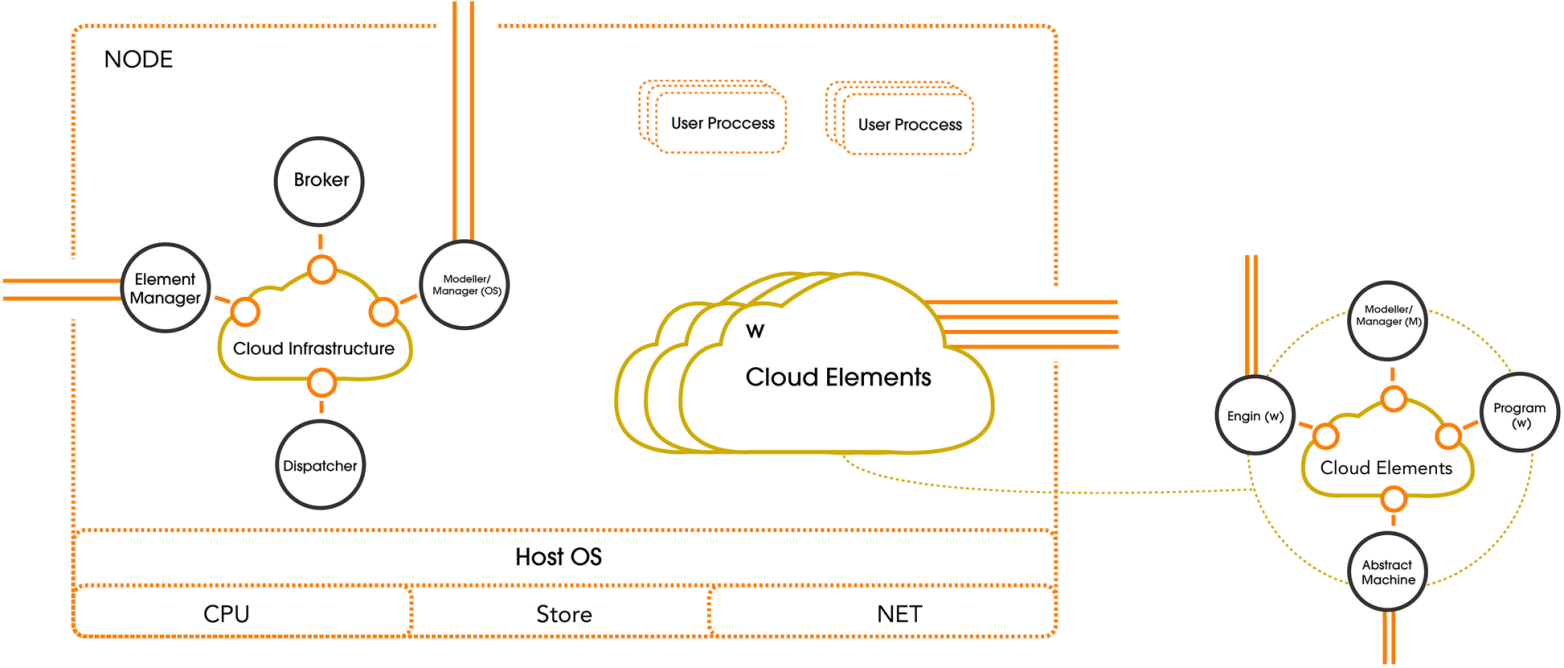
An illustration for the proposed designed nodes’ components between the cloud infrastructure and cloud elements that was proposed in [24]

Kirby et al. [24] have described the concept of ad-hoc cloud systems first, they have suggested the idea of implementing ad-hoc-cloud systems to be configured incorporates through better usability of the existing resources, minimizing net energy usage, and empowering enterprises to run their own ad-hoc cloud system. Their experiment presents one strategy to overcoming the main research and project obstacles of ad-hoc cloud system. However, the main issue was to deal with intermittent hosts and limit the effect on non-cloud operations. A modeler/manager module is implemented on the VM in every cloud component shown in Fig. 1 below. This monitors both the host's resource utilization and execution. The host-side counterpart checks the cloud element's effect on the host; tasks were assigned to nodes using both ‘Broker’ and ‘Dispatcher’ architecture. Module for the ad-hoc computing system design. The presented systems differ significantly in scheduling strategies, QoS assurances, and the mechanism in which the incorporate reliability through an un-stable infrastructure, among other aspects [25].

**Fig. 2:**
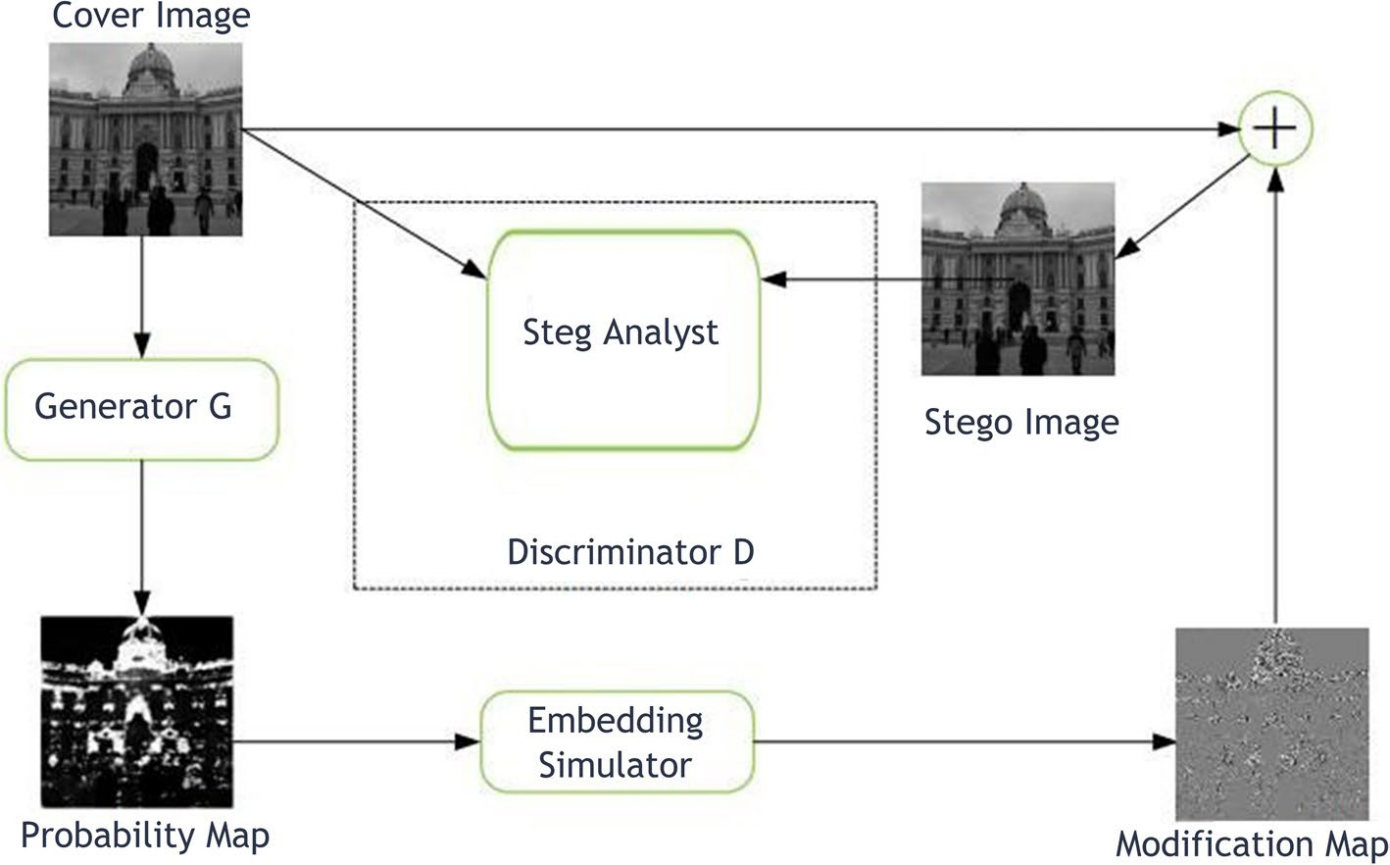
The (Alteration Probability Map) looked at in [26] was made with the “Automatic Steganographic Distortion Learning” method

Chaumont [26] the authors have presented a full practical evaluation regarding the utilization of deep learning in steganography and steganalysis. The study was limited to the applied proposed techniques between 2015 and 2018 in order to provide new future directions through highlighting the limitations of the reviewed techniques. The main components of CNN were deeply discussed from the perspective of both time and memory. Multiple techniques were discussed in detail to get at the roots of the idea of the recent proposed methods of using steganography with deep learning methods. The study has concluded that there were still limitations and challenges that remain regarding the experimental phase of the proposed studies, as there were a lot of restrictions that may prevent applying the previous studies on a large scale. As shown in Fig. 2 below, an example of a framework that represents one of the discussed early deep steganography approaches, which was the (Automatic Steganographic Distortion Learning), from which they can get the (Alteration Probability Map). Finally, based on a thorough comparative analysis, the authors concluded that future works should specify enhanced algorithms to improve the efficiency of deep learning networks with various types of steganography.

**Fig. 3:**
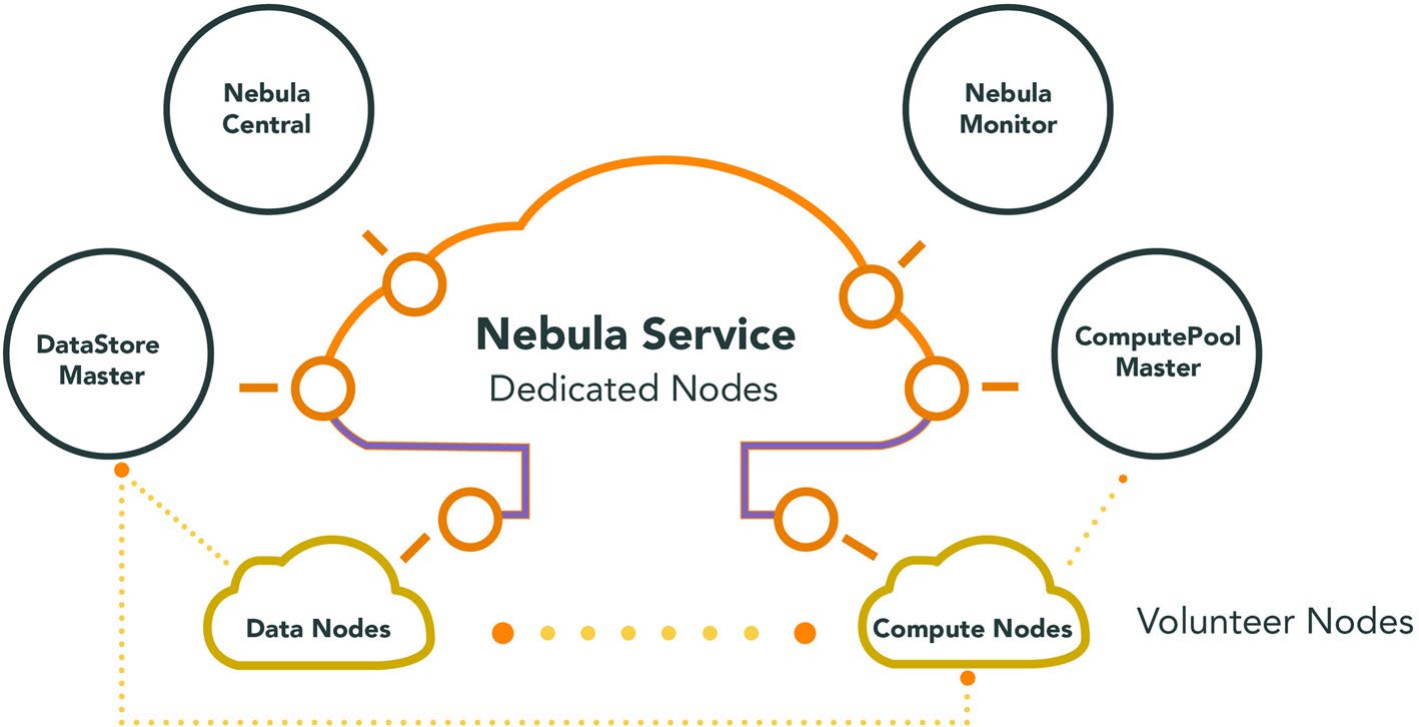
The designed ‘Nebula Service’ system structure and its connections between ‘Data Nodes’ and ‘Compute Nodes’ as proposed in [27]

Chandra et al. [27] have studied various techniques for implementing clouds with the usage of volunteer resources. They describe the difficulties of constructing clouds (Nebulas) from erratic resources through tackling similar sort of issues with different methods as shown in Fig. 3 below. Therefore, the distinctions between their approaches were minor. Ad-hoc Cloud Systems, or Nebulas, were dynamic infrastructures that combine features of both 'Volunteer Computing' and 'Cloud Computing'. Various issues were arising an example; a software may be experimental and not require strict system execution assurances. They have proposed two solutions for errors handling, they use replication to run a job on various hosts simultaneously, or do VMs check pointing then restoring those checkpoints on host failures.

It can be as well prohibitively costly for the migration process, especially if it depends on massive volumes of dispersed data. For software deployment, correctly for a set of resources, resource scheduling is required. Larger applications could be distributed on faster servers to decrease the influence of the slowest host on actual system execution; these hosts have to be reliable [28], given that ad-hoc clouds often work on infrastructures with limited host count, the ‘Task Redundancy’ was not used, as using it can reduce the amount of accessible ad-hoc hosts for new cloud workloads. Additionally, it was suggested to calculate the network performance for mitigating the anticipated performance degradation [29].

Weissman et al. [30] described their early practices with a model of the ‘Nebula Theory’, for scattered data- intensive softwares working in centralized infrastructures, the authors reiterate the existing cloud unsuitability through the usage of the global research testbed (PlanetLab+), they compare their prototype with the (Data-Intensive Blog- Analysis) prototype. Using 'Nebula Master', the users have the ability to join the cloud and the admins can monitor and control the system. Compute pool elements control volunteer storage and computing nodes. With more blogs to analyze, Nebula saved time with a data transfer rate of 53% compared to a cloud emulator. When a comparison is made with the (Centralized Cloud) framework, the ‘Nebula’ outperforms the (Centralized Cloud) framework once the failure of a few hosts. Nebula uses task duplication and re-execution to provide fault tolerance. As stated before, ad- hoc cloud systems face both various theoretical and practical obstacles. However, preliminary results that indicate promising indications [30].

Duan et al. [31] They proposed an algorithm based-steganography extraction through using the DNN, this technique had the ability to combine both DCT and ECC in an image. Firstly, the ‘Secret Image’ would be created from transforming the original image that was previously written using steganography approach, through the SegNet DNN paradigm, the classified image is incorporated in the host image. It would not be difficult for altering the host image, and the image quality would not be affected adversely, while the anti-detection characteristic is also strengthened. In addition, steganography capacity is guaranteed; as the DNN framework was used, all what was needed the ability to change the variables in both processes, a) The embedding process, b) The extraction process, with no need for additional formulas to be created, the applied models were employed to be with higher adaptability level in the system [31].

Yi et al. [32] have discussed in their study both a) an example of a cloud service provider (i.e., Dropbox) that utilizes resources from non-dedicated servers, b) a basic prediction methods combined with host rating provide reliable long-term forecasts. The less dedicated servers or cloud services means less costs for the web service providers; as a result, the authors have limited their method to the cloud service field, in which non-dedicated hosts were employed for processing. Non-dedicated hosts have restricted bandwidth and were not always available. Nevertheless, the authors made an assumption that the web service can provide both fault tolerance and redundancy methods in order to deal with extremely volatile non-dedicated servers, the authors study was divided into two parts: Firstly, they have addressed other research issues; to help forecast long-term fault tolerance for non-dedicated hosts, they evaluate strategies to predict short-term fault tolerance through defining a strategy to recognize ideal mixes of dedicated and non-dedicated servers for both cost reduction and migrations. Their results demonstrate that the average maximum and minimum long-term availability forecast error rates were around 22% and 15%. Moreover, with the existence of high-rate non-dedicated servers' number. Increasing data redundancy also reduces the dedicated hosts’ number necessary to achieve availability assurances. The weakness of the proposed ad-hoc cloud is it lacks redundancy and instead reacts to host failures.

It is expected that adding task redundancy for ad-hoc cloud can improve task completion rates. Secondly, they have proposed an optimization strategy to reduce web-service provider charges or migrations. The authors believe dedicated resources can be delivered through a cloud service provider like (i.e., Amazon EC2), therefore every dedicated host cost 10$ / hour, the same goes for Amazon EC2 instance. Data Transferring through a failing non-dedicated host to a further one restores processes. Finally, the authors compare the benefits of employing dedicated versus non-dedicated hosts. In order to use a non-dedicated host's resources, a cloud service provider must first confirm the host's availability using weekly monitoring data, many machine-learning classifiers were mainly utilized to group the servers based on the projected short-term availability [32].

Wang et al. [33] have introduced in their study a unique method of steganography based-Stego images created through DCGANs. From another perspective, CNNs were used to implementing a functional link among both the ‘Secret Data’ and the ‘Stego Images’. Moreover, the CNNs models, which have extraction ability for secret information from stego pictures, was the main contribution of their study. Image steganography can effectively evade steganalysis approaches because of the proposed improved technique regarding the ‘Secret Data’. DCGANs have two obstacles; as they would be used for image steganography. Not all the created Stego images have high quality; as the small size of the Stego image is not meeting the minimum requirement to conceal a data. The study discussed creating a resilient CNN to solve the mentioned obstacles. Error-correction codes were added to this approach; in order to improve the accuracy. As a future work, many advanced algorithms could be proposed to enhance the quality of the approach and to overcome the addressed obstacles.

Mori et al. [34] have describes SpACCE's as an ad-hoc cloud infrastructure dedicated for software sharing. Their goal is to implement a cloud environment through the usage of an ad-hoc system named 'CollaboTray', it can move to another network node at any time (i.e. Microsoft Office Package). The server can re-locate in case the node presently hosting it becomes overloaded or if the service supplied to clients suffers. If a software needs additional resources over the server capability, other clients may be turned as servers; because their project implementation is based on ad-hoc concept, the main targets were similar: how to efficiently coexist through user procedures, communicate over dynamic hosts, in addition to component migration. Their findings reveal that a server's performance can suffer if only 40% accessibility of the CPU. Consequently, resource heavy apps cannot use 'CollaboTray'. For the migration process of 'CollaboTray', it should be first shutdown, and then its current state is moved to another node. Finally, it would be restarted. However, 'CollaboTray' was not using virtualization, so the system's security is compromised in case the server has moved to a suspect node. In case the server has moved to an un-reliable node, the software performance would be affected. The proposed ad-hoc system implementation phase have addressed all these issues, in addition to providing additional capabilities (i.e. monitor – schedule). From their perspective, the cloud computing represents a business model that limits its scientific software. A unified cloud concept is proposed as a replacement to data centers. It's called Cloud@Home since it was comparable to 'Volunteer Resources'. A 'HybridCloud' permits users to resources’ subscription from an 'OpenCloud'. Those two cloud frameworks can be utilized separately, or linked to further cloud system, respectively. Data privacy along with secure communication protocols offer security for centrally managed resources and data. These were among the significant challenges identified by the authors in their research.

Zhang et al. [35] have proposed a full framework of using DNN with steganography, a better understanding of how DNN-based deep hiding operates through contrasting it with the DDH utilized and the newly suggested UDH. For example, if you want to hide a single image in another, you can do it with this understanding. The container picture can be utilized to give varied content to various users based on their practical demands while we demonstrate the capability of retrieving distinct hidden images by different recipients. It has become a challenge, with the increment of images/videos which were classified as intellectual property; the "Universal Watermarking" definition was used, as the proposed UDH provides a temporarily solution to for such a problem. UDH can also be utilized to transmit small messages, as demonstrated from the authors experiment; the study proved that the results were promising for hiding an entire image, which significantly increases the potential future works from different directions [35].

Wu et al. [36] build a BOINC-based private cloud for similar and distributed replication. With BOINC system as a dispatcher, the author's own load-balancing methods were using schedule tasks for nodes inside the system. They mention scheduling and infrastructure observing as crucial components through private cloud platforms, but do not mention the usage of BOINC or the framework their technique based upon. In their view, cloud systems represent business models that limit its scientific software. A unified cloud concept is proposed as a replacement to data centers. It's called 'Cloud@Home' since it's comparable to volunteer computing. The 'HybridCloud' provides users subscribing resources from an 'OpenCloud'. These two cloud frameworks have the ability to be used separately, or linked to further public/private cloud platforms, respectively, they have designed a BOINC-based private cloud for distributed implementation. With BOINC role as a correspondent, the author's own load-balancing methods were mainly utilized for task scheduling to nodes inside the system.

Girardin et al. [37] have designed a software named Legion for generating web portals for various functions, including uploading processes to V-BOINC. As it is done by developing a cloud interface which communicates through a Legion cloud service using SOAP. Therefore, legion creates and maintains redundant data based on the BOINC database, making it difficult to connect with V-BOINC tasks. 'Legion' also needs more libraries for submitting the task to V-BOINC; because 'Legion' conducts so many activities, it is analogous to the reason 'WS-PGRADE/gUSE' did not utilize to submit jobs to V-BOINC. Others that promise to permit 'Job Submission' to V-BOINC were either considered unsuitable for our requirements or have not provided the basic capabilities we require. Therefore, we built a private ‘Job Submission System’ that works with BOINC [37].

Regardless of the limited ad-hoc cloud system success as well as the merger of volunteer computing, mobile computing provides more success and popularity since 2009 [38]. Mobile devices were considered as a 'Resource Poor' elements (i.e. Limited Storage Size, Computation, and Memory Capability), they were as well constrained by both battery lifetime and network connection [39]. Offloading into another remote computational platform would be an advantageous in some instances, such as rendering high-quality images in time the power is limited. Most research focuses on whether executing apps within ad-hoc mobile cloud systems is viable and whether performance advantages might be attained. Success stories vary, and the apparent benefits depend on the software, because of WAN latencies or cloud platforms cannot be properly offloaded [40]. However, some argue that offloading computing through Amazon EC2 can be practical and beneficial regarding latency-tolerant softwares. In order to make the best use of locality, the autonomous mobile nodes set termed cloudlets is proposed, allowing devices to offload duties to extra users [41].

Wengrowski et al. [42] utilized the deep learning algorithms for digital steganography into the photographic realm for LFM, in which the coded images were conveyed via light; to enable consumers for examining displays and digital advertising with their webcams with no broadband connection. Concerning the digital steganography, CDTF has radio-metric effects, which have the ability of altering the image's appearance; as CDTF was trained with a dataset contains about one million images; in order to model these impacts. The outcome represented the system which formed hidden messages which could only be retrieved with extreme precision, and consequently it could not be seen using the normal eye. For each evaluated camera-display combination, the LFM approach had provided higher outcome of BER score than the previously proposed DNN and (Fixed Filter Steganography) methodologies. Both (Camera Exposure Configurations) and the (Camera Display Angles) have no effect on their proposed study, which outperforms all previous studies at the angle of 45 degrees of screen sight. The dataset contains 1 million pairs were collected through using 25 camera and screen pairs for the CameraDisplay. Finally, these potential DNN-based techniques to steganography provides interesting ideas for future works.

Satyanarayanan et al. [43] provide in their research a simulation for a mobile cloud system that strictly resembles our proposed technique without the usage of mobile devices. In their work they, propose the usage of VirtualBox on mobile devices; nonetheless, some studies found VM-based approaches ineffective. They have studied how to shrink VM sizes in addition to the used approach to move them between devices. Our technique of storing pre- configured virtual computers on devices for sending overlays (checkpoints) via a network that equals theirs. However, they did not address some features such as (Schedule – Task Recovery – Mobile Churn).

## Problem statement

The huge amount of data that is shared between organizations and public cloud services makes it more likely that privacy will be broken by accident or without permission. Commonly, normal users are thought to be cloud platform security flaws, information leakage, viruses, or illegal behaviors, and cybercriminals aim to steal cloud infrastructure security weaknesses for financial gain or other illegal purposes [44]. Cloud services can monitor IT systems, but they are difficult to secure. Even though cloud computing raises privacy concerns, this has not stopped its growth or the decline of data centers. All organizations need to reevaluate their system security rules to avoid sending data without permission, losing service, and getting bad press [45]. In addition to cloud services, public APIs expose enterprises to new security concerns. Cyberattacks target cloud infrastructures, and the capability to attack a suspect's system using penetration testing tools on a cloud platform is a frequent tactic employed by cybercriminals [46]. As it is common to confuse the concept of cryptography with that of steganography, auto encoder network (AEN) is the technology used for compressing images [47]. The objective is to safeguard private data that is sent over networks. During the training phase, the network should adjust the compression techniques for secret image data to the lowest levels of the "Cover Image." Several of the previously described experiments utilized Deep Neural Networks (DNN). As a result of the recent positive contributions of deep neural networks to steganalysis, there have been numerous attempts to incorporate neural networks into the actual concealing procedure; in order to choose which LSBs to substitute in an image well with representing the text message in a binary form, numerous studies have used DNNs to determine which parts of the image data should be retrieved [48]. Neural networks were used to figure out the time of encoding for the categorized data, which was spread across the image's bits. Encryption and decryption networks have been trained together so that the hidden image can be found. Since networks have only been trained once, the set of images used for the hide and secret does not affect how well they work. This work has a "cover image" with 8 bits for each color channel. Another "cover image" with N x N x RGB pixels can be used to hide an encrypted image with N x N x RGB pixels [49]. Even though previous studies required that encrypted messages be transmitted with perfect decryption, we relaxed this requirement in our investigation. Regarding both "carrier" and "secret image," compromises would be made based on the quality of the carrier and the hidden image. We briefly discuss the discoverability level of the detected message's presence as an afterthought [50]. The bit rates used were 10% to 40% higher than those seen in previous research, which showed that buried message bit rates (i.e., 0.1 bpp) could be found. Even if a hidden message is difficult to detect directly, a quantitative analysis cannot rule it out [51]. This research's overarching goal was broken up into two stages in order to introduce a comprehensive strategy for

A) Deploy an end-to-end solution based on an ad hoc cloud integrated architecture and then assess the method’s adherence to performance and privacy benchmarks. Create a deep learning-based steganography model for protecting ad hoc cloud-based data and image transmission. Make an ad hoc cloud architecture by bringing services in through unreliable and unreliable networks. The goals of the study, which were to improve both availability and efficiency, suggest that the technology would work best on local area networks. Broad Area Networks (WANs) can be used to test the security procedures. In some cases, the cloud’s ad hoc nature may not work well for highly secure systems. When a host suddenly crashes because of data inconsistencies, it can cause problems for applications, especially those that send information to outside monitoring.
B) Plan the framework of a steganography method that makes use of deep learning. The model implemented should be able to hide a “secret image” of size N by N by RGB pixels somewhere within a “cover image” that retains the same color depth as the original. Second, beyond those studies, it must be able to propose a method for successfully lowering the limits through which the “Secret Image” has supplied without degrading the quality of the original image. The main problem is that both the “Secret Image” and the security of the carrier must be broken.

## Proposed model

**Fig. 4:**
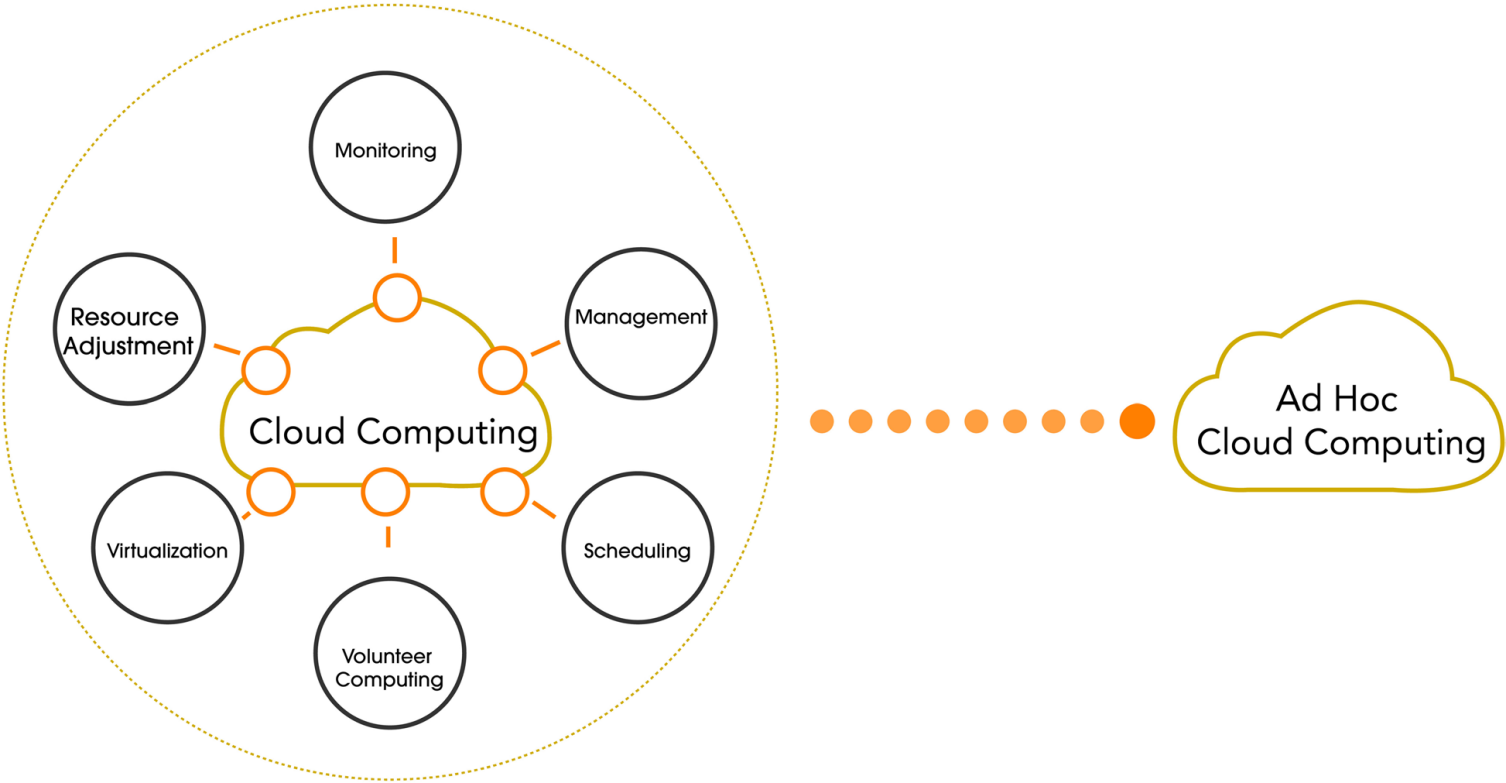
The six main principle features that represent the adapted model of ‘Ad-hoc Cloud System’ model structure from [34]

The proposed design for ad-hoc high-level modules that were used for implementing an ad-hoc cloud system. was built based on the V-BOINC, therefore, we inherit several of V- BIONC features were inherited and an initial client- server architecture [52], the architecture of the ad-hoc cloud was discussed in-detail in this section, then comes a comprehensive design for the implemented prototype. Figure 4 below represents the high-level fundamental components that is a part of ad-hoc cloud computing system structure.

### Ad-hoc cloud computing system architecture

Ad-hoc computing systems should be set up according to the following rules so that they do not have the same problem that most cloud computing systems do:

- ○ Ad-hoc Cloud Model: The ad-hoc cloud model derives heavily from both public and private cloud system platforms, hence shares many specifications. As an example, the ad-hoc cloud represents the PaaS cloud that allows multi-tenancy and provide appropriate QoS [53]. The ad-hoc cloud system implementation success depends on creating an ad-hoc cloud model based-on the architecture of the existing cloud platforms. Now we will talk about the implementation mechanism of the presented ad-hoc cloud computing model [54].
- ○ Volunteer computing: The ad-hoc cloud system would not be able to monitor and control a huge number of distributed and unexpected resources. The ad-hoc cloud would include a component that recognizes the host user accessibility to the resources. As the virtualized BOINC platform would be the best choice for the proposed ad-hoc cloud computing system; because it provides these features [55].
- ○ Virtualization: Securing the host resources and procedures, the virtualization importance was demonstrated in the earlier section regarding V-BOINC; as the virtualization features like check pointing might be used for extending the volunteer platform and provide a system with high reliability for the ‘Cloud Jobs’; to provide fault- tolerance [56].
- ○ Scheduling: Owing to the ad-hoc cloud system in-consistency, the reliability feature was considered as one of the necessities for cloud operations to run rapidly; extra scheduling algorithms must be developed that include resource demand, availability, specification and reliability [57].
- ○ Monitoring: Beyond the presented controlling and monitoring approaches by volunteer computer infrastructures, were essential to supply data for these decision schedules. Advanced controlling is also necessary to provide monitoring for cloudlet, which provides administrators to expand cloudlet resources [58].
- ○ Management: With infrastructure management, administrators can undertake cloudlet, troubleshoot issues with single hosts, or perform tasks across several ad-hoc hosts [59].
- ○ Resource Adjustment: The possibility of limiting the cloud process intrusion would decide the viability, while this function could be difficult for appliance in the proposed model; both the basic virtualization technology and open-source tools can provide this function [60].

#### Ad-hoc cloud server

Ad-hoc servers represent the expansion of V-BOINC servers. Although ad-hoc systems as well as V-BOINC servers distribute VMs to volunteer servers, the (Ad-hoc Cloud Host) can achieve more. It can accept jobs from ad-hoc cloud users instead of V-BOINC or standard BOINC servers [61]. We configured the hosts in ad hoc cloud workloads and VMs that were near optimal in terms of resource load (current usage) and reliability.

- Transmit commands to ad-hoc
- Control and configure the system quickly.

**Fig. 5:**
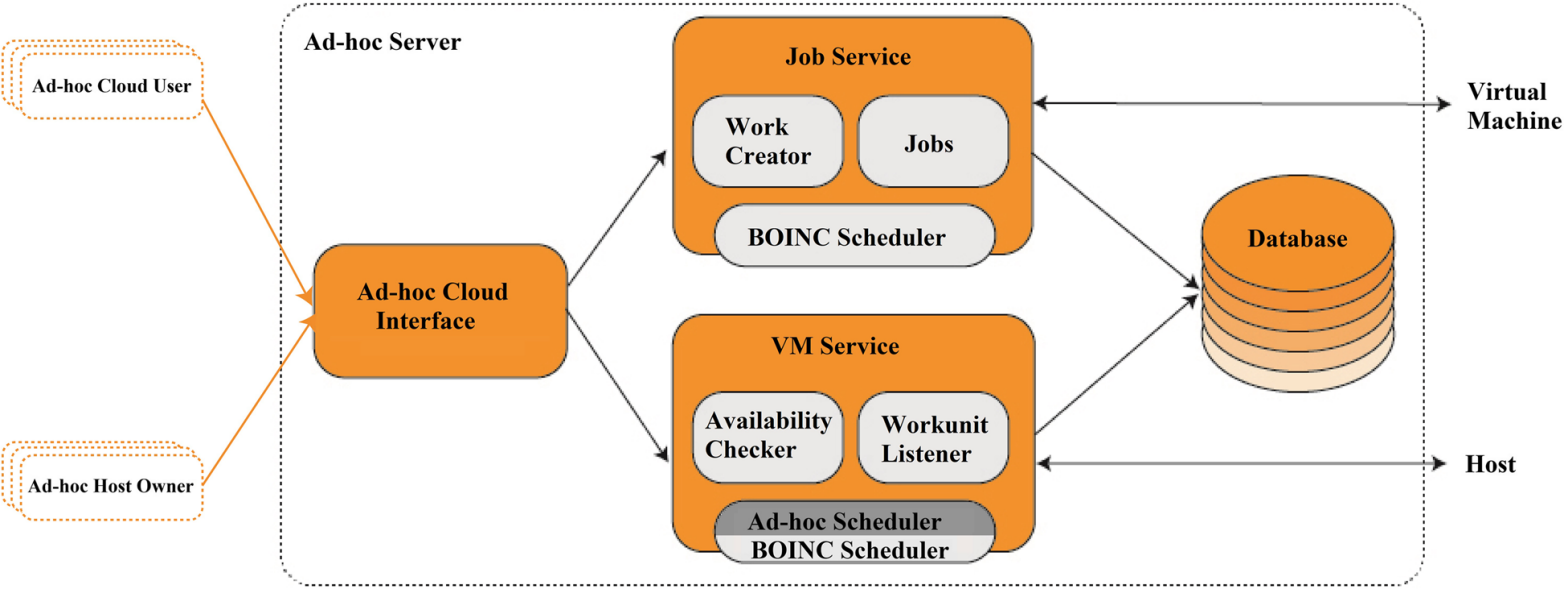
A diagram that focus on the ‘Ad-hoc Cloud Server’ four main components design between the ad-hoc user and the VM host

These new features transform the V-BOINC infrastructure into an (Ad-hoc Cloud System) as these two aspects were previously available through two BOINC features (VM Service and Job Service), in contrast to the V-BOINC server, which serves volunteers via a BOINC node scheme named V-BOINC. Figure 5 illustrates the structure of the ad-hoc cloud host.

The 'Job Service' can obtain 'Cloud Jobs' through the ad-hoc hosts and register them with BOINC. After registration, the 'Job Service' must notify the 'VM Service' that the 'Cloud Job' is ready for execution through the guest on the host. The 'Ad-hoc Cloud Interface' as well can provide both users and admins to control their BOINC users. The tool 'VM Service' would be distributed through ad-hoc hosts. Determining near-optimal ad-hoc migrations of hosts and virtual machines, sending instructions to both ad-hoc hosts and ad-hoc guests, and monitoring/controlling the overall system status. Ideally, both 'Cloud Jobs' and 'VMs' might be handled from only one V-BOINC scheme. Nevertheless, this might not be achievable since there is a need to discriminate the two entities; as it has more functions than the standard BOINC servers do, although its architecture to the standard BOINC servers is comparable.

#### Ad-hoc cloud client

**Fig. 6:**
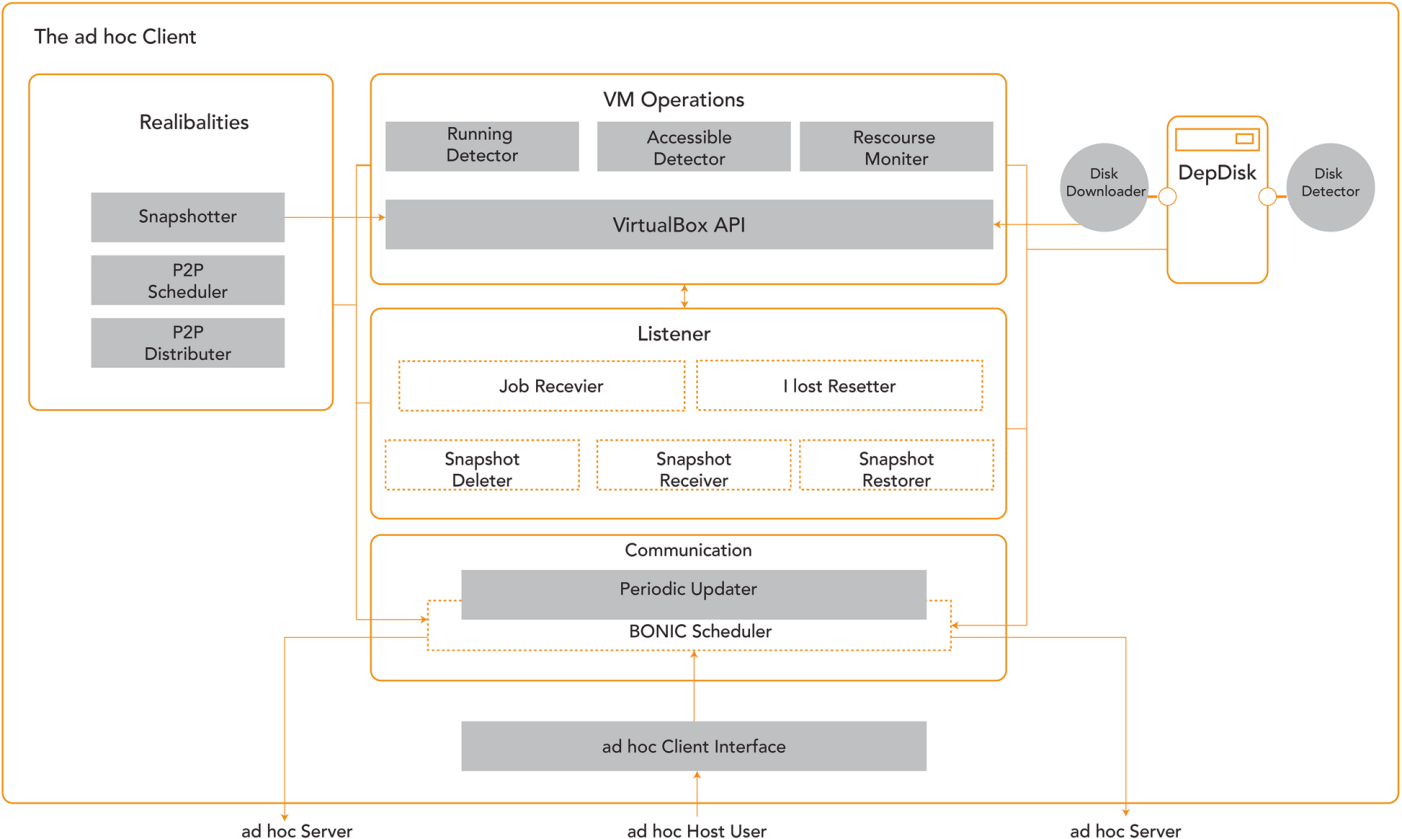
A description of the (Ad-hoc Cloud Client) through its four main components: VM Operations, BOINC Scheduler, DepDisk and Reliabilities

The ad-hoc cloud system was represented through the V-BOINC client add-on, as shown in Fig. 6 below. Furthermore, for the execution of volunteer applications on the VM volunteer host, the ad-hoc cloud client must provide a reliable environment for the 'Job Execution'. Unlike a conventional V-BOINC client, it has the ability to:

- Create regular VM checkpoints.
- Transmit checkpoints to the optimum ad-hoc hosts’.
- Ad-hoc hosts collect the VM checkpoints from all the ad-hoc hosts and restore them from terminated or unsuccessful.
- Control both ad hoc and other users.

The structure of an ad-hoc client is more difficult than the V-BOINC client because of the vast number of functions implemented through the V-BOINC client.

The main ad-hoc client modules together represented all the ad-hoc cloud’s user interface, connectivity, listener, and reliability. The GUI (i.e., BOINC Manager) controls the ad-hoc host's membership in the ad-hoc cloud. Meanwhile, the Listener component awaits any instructions provided through the ad-hoc server; this can involve functioning on the VM through the (Virtual Machines Services Component), which handles all features of the VM-VirtualBox connection.

#### Client and server interaction in the ad-hoc cloud system

Both the server and the host in the ad-hoc cloud system communicate with the usage of the BOINC connection methods [62]. However, V-BOINC have been modified to allow customized data flow among the server. For instance, for upgrading the server on the VM status, BOINC enables client-server connection through sending XML messages that were interpreted locally by the received object; the host's information is transmitted to the server [63]. The authenticator identifies the (Host, BOINC version and Platform Type), the V-BOINC was restricted from consuming no more than the accessibility of 90% from the memory by the volunteer users, which provided in the volunteer host idle state.

#### Practical evaluation analysis of the (ad-hoc cloud system)

A comparison was made between, a) the operation of the (Ad-hoc Cloud System), b) the process of the (V-BOINC Server), both client and server tasks and communication techniques differ in the ad-hoc cloud system; as the ad-hoc cloud host has to:

- Install the ad-hoc client, after that automatically requests a VM from the ad-hoc server.
- The ad-hoc host gets a VM in addition to the decompression script.
- The VM is installed at a time it has work to achieve. In contrast, once there was an established connection with the ad-hoc server, the VM would be quickly deployed and ready to operate.
- Various VM images cannot be used if there were not enough cloud tasks to fill them. In this condition, the user in the ad-hoc cloud can execute a job into the ad-hoc cloud server, and then the ad-hoc cloud client would be ready for the execution.
- In case the job includes dependencies, the ad-hoc server then might download the ‘DepDisk’ sent through the user, V-BOINC’s actions were followed by other procedures.
- The ‘DepDisk’ is attached in this state and the new VM disk is established and connected in case one could not exist.
- The new VM should be created, then assigning the main function task for both ‘Cloud Job’ and ‘Cloud Data’, which runs the job and shows the results for the server.

Basically, despite the similarities in client-server topologies, the individual components of both 'V-BOINC' and ‘Ad- hoc Cloud System' were very distinct. With these features, V-BOINC has evolved from an ordinary virtualized volunteer infrastructure into an ad-hoc cloud system, replacing BOINC and its virtualized volunteer scheme.

#### V- BOINC submission system

The BOINC, the application that the volunteer hosts were utilizing, was generated statically prior to the server distribution; the V-BOINC platforms were then brought to the ad-hoc cloud server, following which BOINC authentication was performed. The first jobs approved by WS-PGRADE [64] were those submitted over the "Grid Network". It permits multiple connections to both the 'Access Grid' and the 'Desktop Grid' while requiring scripting for software implementation on the infrastructure. In addition, the workflow-based graphical user interface 'WSPGRADE/gUse' supports the 'Apache' server-based Liferay portal; the configuration can be performed on a local or distant host. To facilitate job submission, WS-PGRADE leverages 'DCI-Bridge,' a technology that enables standardized computing infrastructure accessibility [65]. WSPGRADE/gUse is compatible with BOINC, but it is not a component of BOINC; rather, it is a service that communicates alongside BOINC. Despite its reputation in the scientific community and the availability of multiple computing platforms, integrating 'WS-PGRADE/gUSE' using an ad hoc approach may be difficult. For instance, 'WS-PGRADE/gUSE' must be configured on the local host. Create a unique web entry point for sending work to the ad hoc cloud system. This obtains more libraries and packages (i.e., Liferay portal project [66]). In addition, the system 'WS-PGRADE/gUse' with some non-required features, such as 'WS-PGRADE' and 'DCI-Bridge', can be used to submit work for future research on the ad hoc cloud system platform.

#### Ad-hoc cloud system GUI

**Fig. 7:**
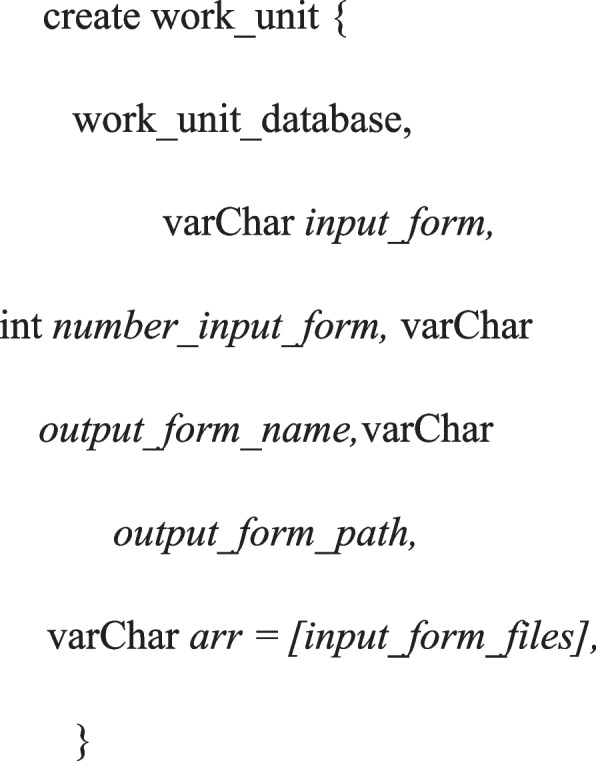
The main parameters used for a work-unit implementation in a V-BOINC system

The graphical user interface of the proposed ad-hoc system is based on the V-BOINC interface. A user's online account enables the modification of volunteer user preferences and the tracking of the status of existing or previous work [67]. To enable using it with the BOINC basic interface, testing additional software types was delegated as future work. As our testing is limited to Ubuntu Server 14.02 [68], the ad-hoc cloud user can choose from a wide variety of operating systems to run a variety of applications in different circumstances. Users can peruse V-home BOINC's page, which lists tasks, to acquire extensive information about the work. After a user uploads and submits a 'DepDisk' file, it is stored in the jobs/directory of the 'Job Service' project [69]. 'Sprite.App' and 'Sprite.Data' were renamed along with the software and other records/files within each numbered directory in accordance with the proposed method. With the use of BOINC, a daemon program known as "Task Creator" [70] is employed; it is mostly used for registering applications and plugins. As soon as a new piece of software is submitted, it is split by conceivable entities into software and input files. Using this data, an XML file describing the properties of a job, including the program and its input files, is generated [71]. We assume that the results of any software are sent to a single output file for testing; hence, we construct an output form file containing the software's results. Using BOINC's "Create Task Function," these forms register the software with BOINC; Fig. 7 depicts "Method Call.

The method generate work accepts the 'DB Work' unit object as its first argument, which describes the work unit or ‘Cloud Task’ to be implemented. For instance, the highest disk / memory utilization rate; because the cloud job then can launch a VM that can only consume so many ad-hoc cloud system resources, we let the work unit use 100% of every virtual resource. The next three arguments represent the input form's contents and the output form's term and destination. The ‘DepDisk’ should be transmitted to the ad-hoc host just before the VM working; to prepare an (Ad- hoc Cloud Host) for working before the VM execution phase, there is a need for using the ‘Task Creation’, and ‘SCHED CONFIG’ object with data related to the V-BOINC project [72]. Upon a successful function implementation, the BOINC work-unit was established and saved in the ‘Job Service’ DB, work-unit ID and logs were saved as well, then the ‘Ad-hoc Cloud Guest’ receives the work-unit from the ‘Job Service’ DB.

#### Job scheduler

Job Scheduling is a process that could be implemented through the Ad-hoc Scheduler when a job is submitted to BOINC. However, due to the separation of the "VM Service" and the "Job Service" schemes, the "VM Service" is unable to determine whether the "Job Service" has received a job or not [73]. As a result, the (Work Unit Listener) Daemon was added along with the 'VM Service' that scans for any new work-units in the 'Job Service' DB, as the 'Work Unit Listener' periodically checks the database. The 'Work Unit Listener' notifies the 'Ad-hoc Scheduler' about newly-added 'Job Service' work units. The 'Ad-hoc Scheduler' can begin searching for a suitable 'Ad-hoc Cloud Host' to create the 'Ad-hoc Cloud Guest' based on the 'Cloud Jobs' that exist and are awaiting assignment into 'Ad-hoc Guests'. If the ad-hoc cloud computing system has enough ad-hoc cloud hosts to support cloud 'job scheduling,' this decision was influenced by the 'Ad-hoc Cloud Host's accessibility, requirements, resource demand, and performance. The typical V-BOINC server scheduler can be represented as a (Bag of Tasks) strategy in which a job is simply dispatched to the 'Volunteer Host' [8], along with the necessary requirements (i.e., memory and disk space) for completing the project within a predetermined period. V-BOINC runs a task on many volunteer computers and then compares the results to make sure the task was done correctly.

The (RIDGE System) is a consistent platform [74], which considers a) performance, b) behavior, and c) reliability. It has been created based on the V-BOINC for measuring the ideal task redundancy level. The normal ‘V-BOINC Server Scheduler’ enhances every task redundancy standard statically [75]. RIDGE outperforms the normal ‘V-BOINC Scheduler’ based on the task throughput and might reduce the task completion time through dynamically modifying redundancy levels. This is based on the recent condition of the ‘Volunteer Infrastructure’. Though the ad-hoc cloud computing system might not use the ‘Job Redundancy’ for providing reliability, there were various approaches to calculate the system reliability, as it was previously discussed and compared in other studies [76–79]. Algorithms that accurately reflect volunteer host standing can help to confirm that the volunteer host set cannot be combined to upload erroneous results. Some schedulers have been proven to boost job accuracy while reducing task completion time. Moreover, the "Reliability Schedulers" can forecast future volunteer host availability to detect the time in which the volunteer chores should be executed. The ‘Volunteer Task’ can be broken down into reduced variable length subtasks and distributed across multiple volunteers. In case of matching each volunteer host's performance to the sub-resource task's requirements, the overall job completion time can be lowered in many circumstances. Volunteer hosts can be graded depending on the expected download time for data-intensive apps working within the volunteer resources. After that, the ‘Volunteer Task’ is assigned to the volunteer host along with the fastest download time. Subsequently, the 'Volunteer Task' is allocated into the 'Volunteer Host', scheduling can alternatively be reliant on White box/Blackbox methods, where a lot or little regarding the software is identified before execution. Jobs could be scheduled for the nearest ideal hosts depend on the resource requirements, then the nearest optimum host with the needed resources would be chosen. End-user criteria (i.e., Budget Cost and Performance Management) can also have an affection for the scheduling decisions, while some other decisions could be based on: a) computation time reduction. b) provider profits enhancements and c) the required SLAs appliance.

Previous papers have studied the potential developments to the used *'Scheduler'* in the implemented design for the ad-hoc cloud system [76, 80–82]; as the reliability, improvement of the scheduling plan would affect:

- The overall performance of the *‘Cloud Jobs’*.
- The total time required for completion
- Increase in task throughput

#### Availability

The ‘Ad-hoc Cloud Server’ keeps a group list of accessible hosts defined by the 'Availability Checker Daemon' introduced into 'VM Service' project. The 'Availability Checker' occasionally checks the VM Service DB to regularly check the time in which an ad-hoc cloud client last contacted the server. It is available if the client polled the server within the last 3 min. Ordinary BOINC clients can communicate with the server for job. In most circumstances, despite being accessible to run software, a ‘V-BOINC Client’ might not be able to communicate with the ‘V-BOINC Server’ for lengthy periods. A 'Periodic Updater' module has been installed into the client to check the server connectivity per minute. The 'Periodic Updater' is built as a '*pthread*' generated in a time that the client was created; this time period is saved as a log in the project server DB, that notify the 'Availability Checker' in case any client in the ad-hoc cloud system has enrolled within recent two minutes. They were inaccessible if they have not been polled. Consequently, 'Ad-hoc Scheduler' scans the 'VM Service' DB for any available hosts.

#### Host hardware requirements

Formerly, accessible ad-hoc cloud hosts were assessed in order to check if they could physically perform both ad- hoc cloud guest. Assuming both a cloud task and an ad-hoc cloud guest utilize fair amounts of resources, we cannot identify how many resources would be needed before the execution. Consequently, we suppose every ad-hoc host has 16 GB of RAM, and 80 GB of storage. It is conceivable to compare the consumed both times and resources of previously run 'Cloud Jobs' to anticipate both times and resources of a newly submitted 'Cloud Task'.

It is difficult to identify if a cloud work, before its compilation, shares features with the earlier compilation. This task merits further research; when launched, the V- BOINC client immediately records the volunteer host's resource status. These resources have restrictions and might be configured by the user options in the V-BOINC client and 'Volunteer Software'. The ‘Cloud Scheduler’ observes the resources which the ad-hoc visitor or cloud job possibly access. Ad-hoc hosts, which might not be able to meet the resource requirements, were eliminated from consideration. OpenStack nova-scheduler does something similar by calculating acceptable servers for VM placement using the filters (Core – RAM – Disk) filters.

#### Host resources

The supplementary ad-hoc cloud hosts' resource loads were recovered, this can happen through adding (Ganglia), “which is a scalable, distributed monitoring tool for high-performance computing systems” [83], this tool would be an addition for the client in the ad-hoc cloud system, the host user might not need to setup ‘Ganglia’ independently after the client setup in the ad-hoc cloud.

The ‘Ganglia gmond daemon’ gathers data form the hosts (CPU, Memory, Disc, and Network Usage) in the ad-hoc cloud system. Network utilization could be valuable in identifying which ‘Cloud Jobs’ can particularly fit for the ‘Ad- hoc Cloud Server’. ‘Ganglia Daemon Tool’ collects the ad-hoc hosts’ data in *rrd* files. RRD Tool was utilized to be able to provide ‘Resource Loads’ [84], which collects the CPU requirements for every 15 second interval throughout 120 seconds. The ‘Ganglia’ averages monitoring data every 15 seconds in normal state, but we average load every two minutes to smooth out real-time oscillations and offer a good sense of current demand. In case the ‘OpenStack Scheduler’ is incorporated for the ‘Ad-hoc Scheduler’, the nova-scheduler has started selecting an accessible ‘Ad-hoc Host’ that has the minimum requirement for working, ‘Ad-hoc Host’ processes not preferred to exceed more than 65% of the CPU that has only RAM of 1GB.

‘Ad-hoc Scheduler’ uses the above output to assess in case the existing load is suitable for both ‘Ad-hoc Guest’ and ‘Cloud Task’ implementation. It also stores prospective ‘Ad-hoc Hosts’ DB entries which could be utilized to conduct pending cloud jobs. The ad-hoc hosts should provide the minimum requirements; in order to provide the minimum accepted performance criteria. For instance, the ad-hoc server with 640 MB RAM (Lower than the 1 GB requirement), meeting our 512 MB minimum accessible memory requirement, but the low possibility for accessing greater resources could not provide the 'Cloud Job' the needed performance in a time that more resources were required. Consequently, the 'Scheduler' filters the servers in the ad-hoc cloud system depending on both 'Resource Loading' and 'Hardware'.

#### Ad-hoc cloud host reliability evaluation

Owing to the unpredictable nature of ad-hoc cloud systems, from which systems can fail or shutdown at any time, host reliability must be considered, its reliability was determined by five factors:

- ‘Cloud Jobs’ pointed to ad-hoc host earlier.
- Ad-hoc hosts overall completed ‘Cloud Jobs’.
- User Errors.
- Guest Failures.
- Host Resource Loading.

Furthermore, any type of software/hardware issue that prevents the client from working (i.e., Kernel Panic) can cause host termination; the guest errors include configuration, installation, processing, and shutdown failures; the ad- hoc cloud server monitors reliability factors (a) - (c). BOINC automatically saves the allocated number for ‘Cloud Jobs’ per every ‘Ad-hoc Host’ in the ‘Job Service’ DB. The ‘Ad-hoc Scheduler’ should be requested from the ‘Job Service’ database. VM Services have an ‘Availability Checker Daemon’ that could terminate or fail any ‘Ad-hoc Host’ after being without activity for a couple of minutes. A cloud job's ad-hoc client monitors reliability of factors in previous mentioned points (d) and (e). Timeouts can identify virtual machine configuration errors such as VirtualBox registration failures or DepDisk failures. The 'VBoxManage' API polls the ad-hoc cloud guest each 10 seconds to check the working operation, to ensure that non-operational ad-hoc cloud guests were identified fast and efficiently. The running VMs method returns a set of VMs that were still running.

‘Ganglia Monitoring Tools’ provide functions for controlling and monitoring the ad-hoc host's existing resource overloads [85]. It monitors non-BOINC software’s total CPU consumption and suspends BOINC if their overall CPU utilization exceeds a threshold configured through a volunteer user. Ad-hoc cloud host resource utilization can influence reliability in case whatever monitoring approach is used, in case the host might be substantially exploited by the ad-hoc cloud host processes [86]. Consequently, the (Cloud Job) performs poorly and can have a long time for finishing. The ad-hoc cloud client notifies the ad-hoc cloud server regarding any failure that occurs to the ad-hoc cloud guest. The ad-hoc cloud server has the detection ability of any type of poor performance regularly through the host, the reliability level of an ad-hoc host can be measured at a time in which:

- The ‘Ad-hoc Cloud Job’ has completed its task.
- The ‘Ad-hoc Cloud Guest’ stops working.
- The ad-hoc cloud host has not polled for 2 min.

#### Decision procedure

The prospective execution candidates list has been created based on ad-hoc cloud host (Availability, Hardware Specifications and Existing Resource Demand). This list would then be ranked with every 'Ad-hoc Cloud Host' reliability, the 'Ad-hoc Cloud Scheduler' chooses from a list the most dependable host to allocate the ‘Cloud Job’.

During the schedule process of *n* number of 'Cloud Jobs', the first *n* number of users would be selected, through this way it can be confirmed the reputation level of ad-hoc cloud hosts with the existence of enough resources always have work to do. This is a primary scheduler, which has a possibility for improvements as can be shown as an example in Table 2. For instance, assigning a single 'Cloud Job' to 'Ad-hoc Server'; because building a complicated 'Job Scheduler' can be beyond the focus of the proposed work, the performance assessment and potential inclusion can be left for an upcoming research work.

**Table 2:** An example for individual ‘Candidate List’ in 'Ad-hoc Scheduler’

AdhocHost ID	Reliability Rate	CPU	Memory	Disk Space
14	96	37%	591 Mb	401 Gb
93	77	81%	1.8 Gb	967 Gb
22	58	96%	5.7 Gb	150 Gb
3	39	38%	854 Mb	13 Gb

#### Cloud job working mechanism

In this part, an illustration for the selection process of the ‘Ad-hoc Host’, it was configured for making the necessary procedures to let the 'Cloud Job' to process in the VM. Since both conventional BOINC and V-BOINC represent volunteer infrastructures, the host can control both implementations. Therefore, BOINC clients rarely get notifications from servers unless they ask for them. Ad-hoc servers can communicate with users with no need for waiting for users in order to begin communication. A job receiver can receive a task from any host. After that, the ‘Ad-hoc Server’ re- directs the clients in the ad-hoc cloud system to the basic V_BOINC server message provided to V-BOINC clients. The ‘Listener’ then the message would be parsed to identify the action needed to be taken.

**Fig. 8:**
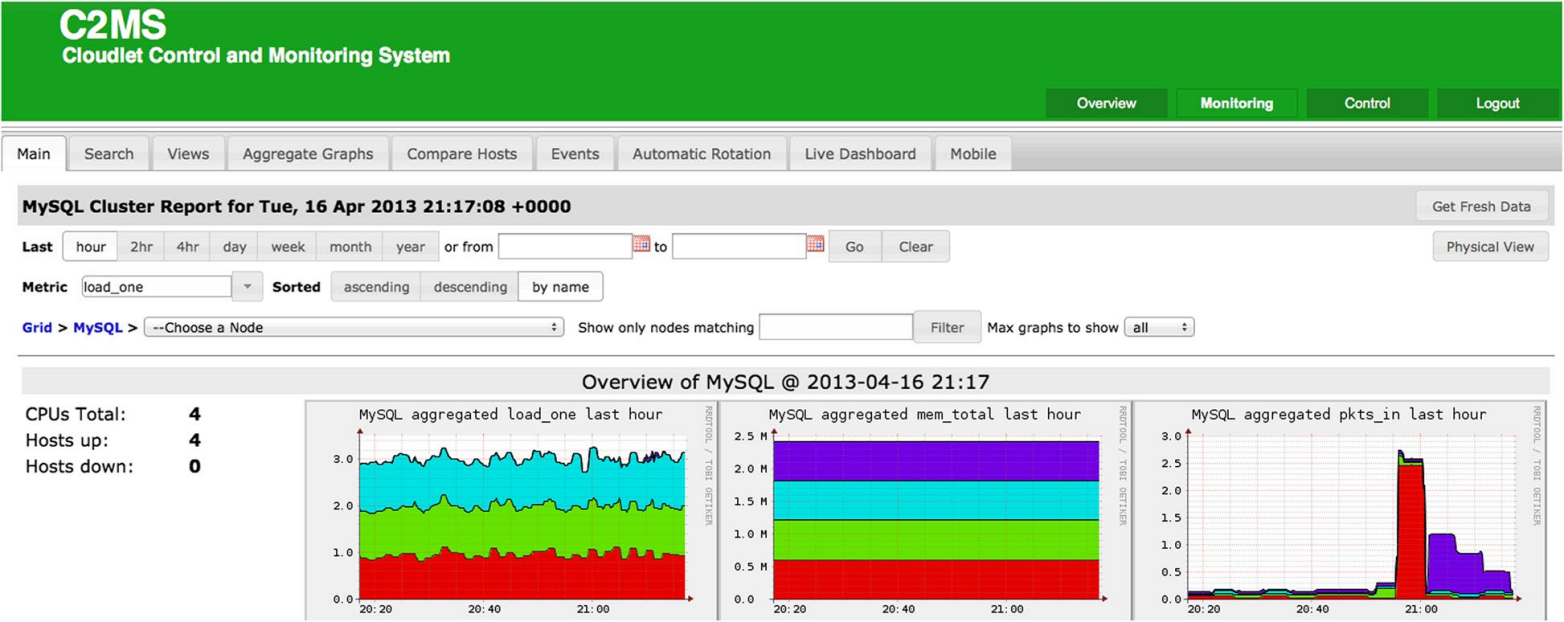
An example of the ‘Cloudlet’ controlling system interface while analyzing SQL live data [4].

The (Job Receiver Listener) should keep the parsed data and start obtaining the 'DepDisk MPI' along with an ad- hoc cloud system, such as V-BOINC, DepDisk will connect to the retrieved VM and start it. The ‘Job Receiver’ after that can direct the VM's ‘V-BOINC Client’ with the aim of communicating with *‘Job Service’* that exists at this URL http://129.205.80.10/Job_Service. The ad-hoc cloud-computing client leverages the VirtualBox API's guest- control function. Despite knowing the work units to give every ad-hoc visitor, the guest sends the ‘Work Unit’ ID request to the server. The (Fault Tolerance) was delivered through the ad-hoc computing system in specific to the client-server interaction. For example, instead of periodically checking for updates for every guest account, we use P2P checkpoints that were assigned to the nearest optimum ad-hoc hosts number, all in the matching cloudlet, to achieve high reliability. The term cloudlet refers to a group of ‘Ad-hoc Guests’, which share software requirements and 'DepDisk', an example for the data analysis interface through the cloudlet within the process can be illustrated through Fig. 8 below.

**Fig. 9:**
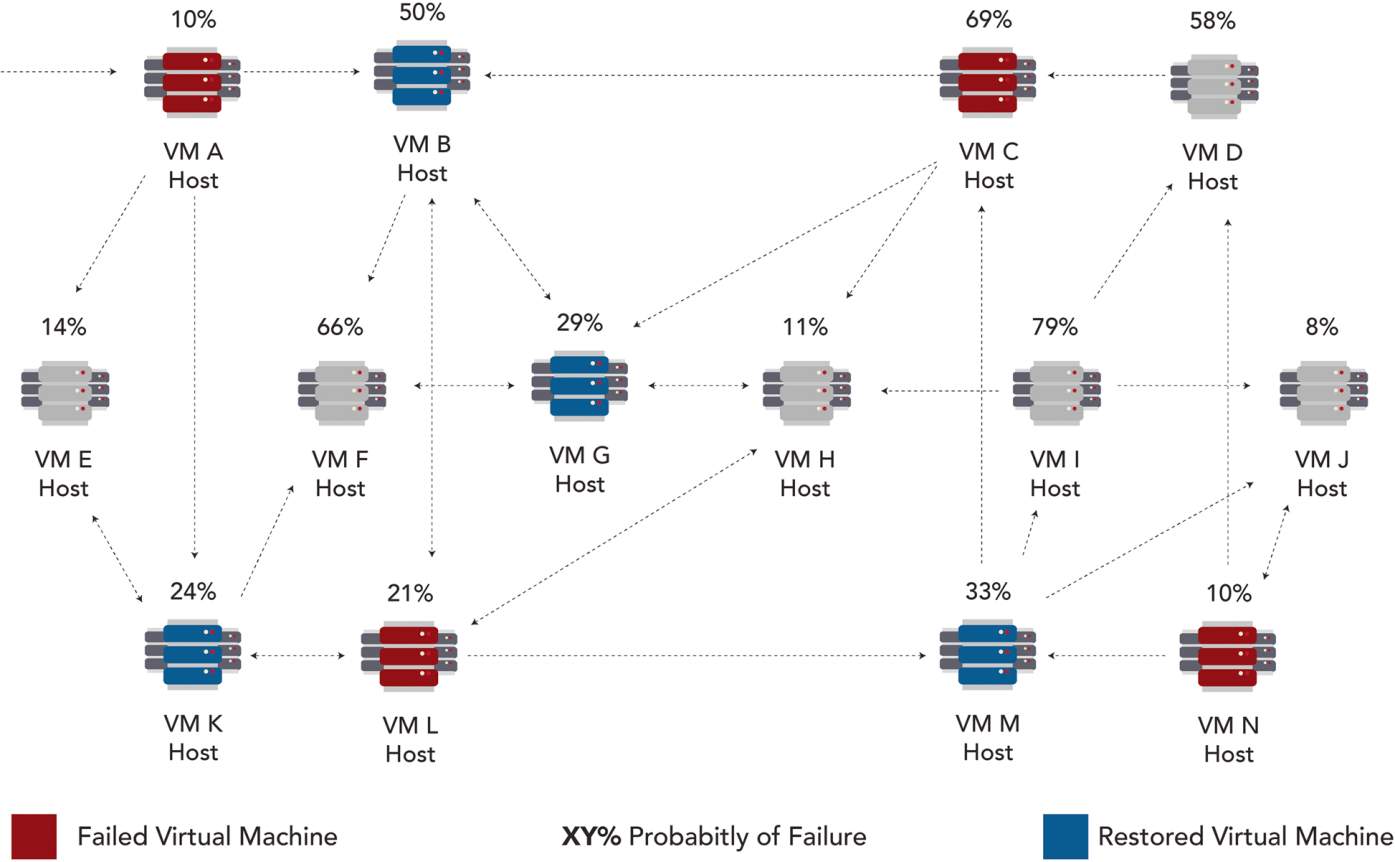
A representation for the peer-to-peer network for achieving reliability, where the failed VMs, restored VMs and failure probability VMs are represented with the colours of red, blue and grey respectively with their rate in percentage

If neither the ‘Ad-hoc Cloud Host’ nor the ‘Ad-hoc Cloud Guest’ fails. Then, the ‘Ad-hoc Server’ notifies the other ad-hoc cloud host for the checkpoint recovery, the peer to peer network was represented in Fig. 9 below, the essentials of the implementations were mentioned in the below points:

- Each of ‘A’ to ‘N’ ad-hoc hosts will include an executed cloud job, or they were waiting for commands on the configured mechanism and set the guests based on the configured method.
- Firstly, the ad-hoc cloud guest ‘A’ will obtain and execute the ‘Cloud Job’. Secondly, to confirm the checkpoints transferring to reliable ad-hoc cloud hosts, the guest should be periodically check pointed.
- The ad-hoc cloud host ‘A’ terminates prematurely, disrupting the guest A’s (Cloud Job) implementation after a period. Because of this failure, it is not recommended to be used in a production environment.

**Fig. 10:**
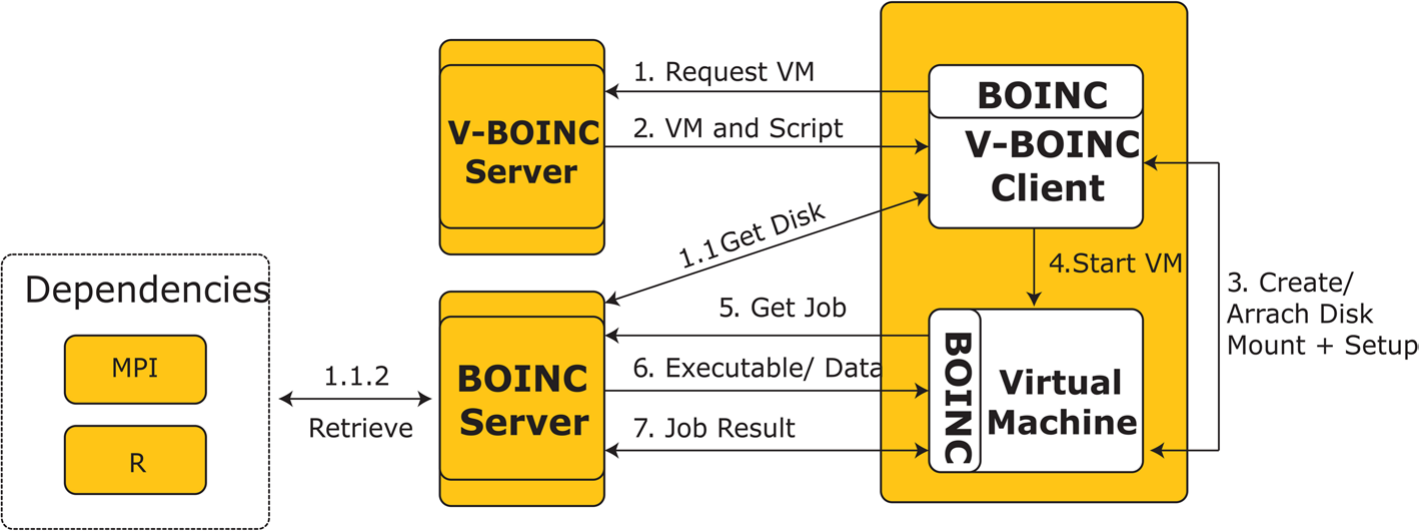
The ‘Ad-hoc Cloud Client-Server’ work flow design starting from data retrieving form the ‘Dependencies’ going through V-Boinc server, V-Boinc Client, Boinc VMs ending with the output from the ‘Job Results’

As previously mentioned, the implementation phase of the (Ad-hoc Cloud System) is mainly based on the Berkeley Open Infrastructure for Network Computing (BOINC) [87], which is a user-server open source solution that was chosen as a framework to harvest the non-used resources from unreliable hosts and integrate them to be part of the system execution. A virtualized scheme of BOINC was created, which is called V-BOINC. It makes the best utilization of virtualization for compiling BOINC features through VMs, starting with V-BOINC servers and ending with BOINC hosts, including administration of the modified host BOINC, which is called 'V-BOINC Host'. It is used for installing the BOINC framework's plugins. Figure 10 below illustrates the overall operation cycle starting from 'VM Request' to 'Job Result'.

**Fig. 11:**
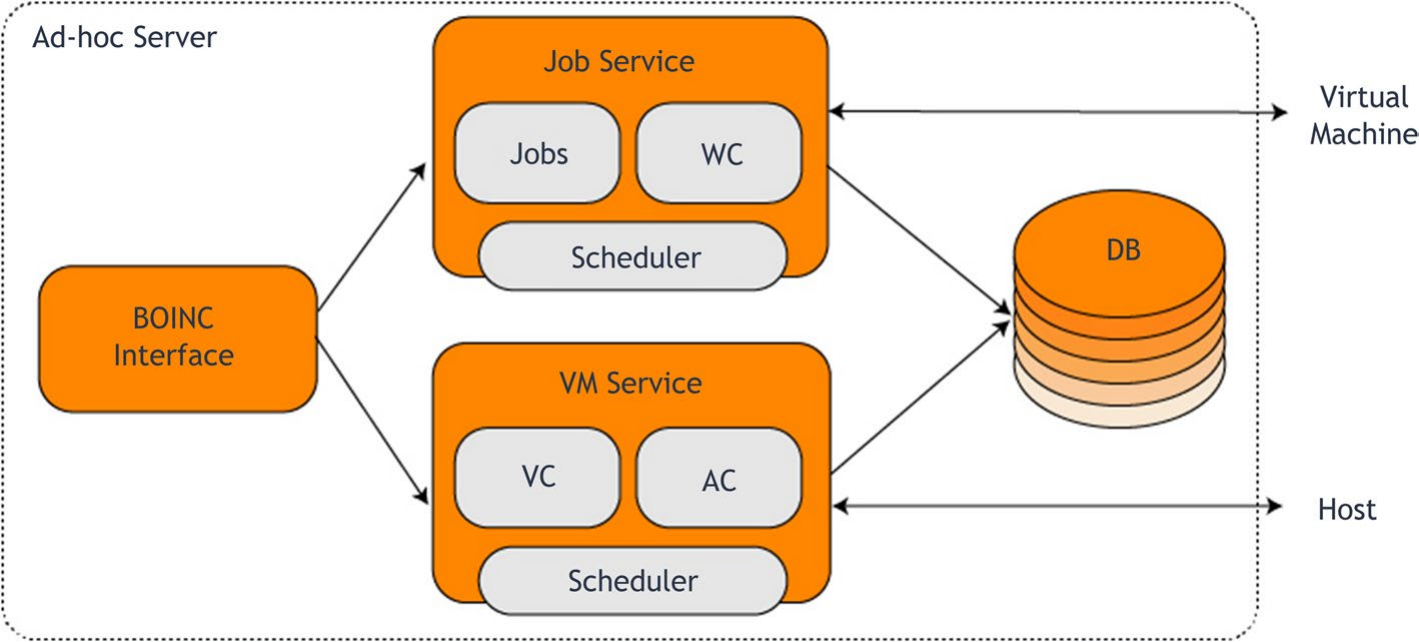
The ‘Ad-hoc Server’ diagram relation between BOINC and the database through both ‘Job Services’ and ‘VM Services’

Firstly, the cloud host imports the data files through the GUI, the overall imported data into the service were located in a folder 'job' inside the 'Job Service' project, then the process will continue through the developed 'Work Creator' tool, this tool identifies whether the import is application or data, along with adding XML information to the files, after that the 'BOINC API' is called for creating the 'BOINC Work Unit'. As the 'VM Service' is notified with the ‘Ad- hoc Cloud Job’ creation through 'Job Service', which allows the 'VM Controller' tool to implement a VM-based volunteer resources regarding the task execution, this operation is represented in Fig. 11 above. Then, the 'job' in the proposed architecture can be executed after a notification is being sent, a 'job' schedule will be assigned to the host with high reliability through the 'VM Service'. Based on certain specifications, the scheduler works depending on:

- The previous overall cloud jobs executed.
- The previous overall ‘cloud jobs completed.
- The failure rate of the host (hardware or software).
- The failure rate of the guest VM errors (configuration, installation, processing and termination).
- The existing available resources.

The evaluation of the reliability measurement for every host through the data transmission rate between both ad- hoc (Host - Server) would be measured as explained in equation 1, in which:

1 $$Host\ Reliability=\left\{\begin{array}{c}\kern1em 0,\kern10.25em if\ NF= CA\\ {}100,\kern9em if\ NF=0\\ {}\left(\frac{CC}{CA}\right)\ast 100\kern5.5em Otherwise\end{array}\right.$$

Where:

**NF:** The overall failure rate of both ad-hoc (Client - Guest).

**𝐶𝐴:** The overall jobs scheduled for the (Ad-hoc Cloud Host).

**𝐶𝐶:** The overall finished jobs through the (Ad-hoc Cloud Host).

The VMs auto-checkpoints were made through the usage of (Snapshot) function in the VMs API. Placing the snapshot files in the auto-assigned destination folder in-which the VMs images were saved. There were various conditions (i.e., VM Settings) that the auto-checkpoint through the VM happens; those configurations represent the hardware settings (i.e., Memory and Desk Size) for each VM. The recent condition to the existing VDI in each VM, this was considered to be done through 'differencing images’ that save all the operations' logs. The recent memory condition, in case the snapshot is saved during the VM processing, the resultant file size depends on:

The assigned memory size for the VM.

- The application memory usability.

Through memory size restrictions, the resultant saved file will be low-sized. However, it can cause some effects on application performance negatively. Regarding monitoring the storage size that is being utilized by the ‘Ad-hoc Cloud Host’, V-BOINC was assigned to erase the unneeded snapshot. For snapshot recovery, the 'Differencing Image' was turned on. As previously stated, the adapted steganography method makes the best use of auto-encoding systems. However, not only a bottleneck for encoding an image is used. Also encoding two images; with the purpose of making the raw image (i.e., container image) has the highest possible similarity with the final image (i.e., cover image). The main network mission was to minimize the error rate through usage of equation 2 as shown below:


2


### Steganography based-deep learning approach

The closest previous presented idea to the implemented steganography in this study was the image size reduction via auto-encoding networks, regardless of two terms were commonly used interchangeably [88–91]. The deep learning model would be trained through a group of hidden images’ data using ‘Cover Image’ parts.

Firstly, the ‘Preparation Networks’ gradually expand the ‘Secret Image’ length towards the ‘Cover Image’ length, dispersing the ‘Secret Image’ bits throughout the full N × N pixels, tests with smaller images were avoided for size considerations, and rather than we focus on real photos. It was crucial to convert the color scheme into further useful elements for concise image recording - that has edges – for overall concealed images sizes. The 'Preparation Network' trains the hidden network in order to extract the ‘Secret Image’.

**Fig. 12:**
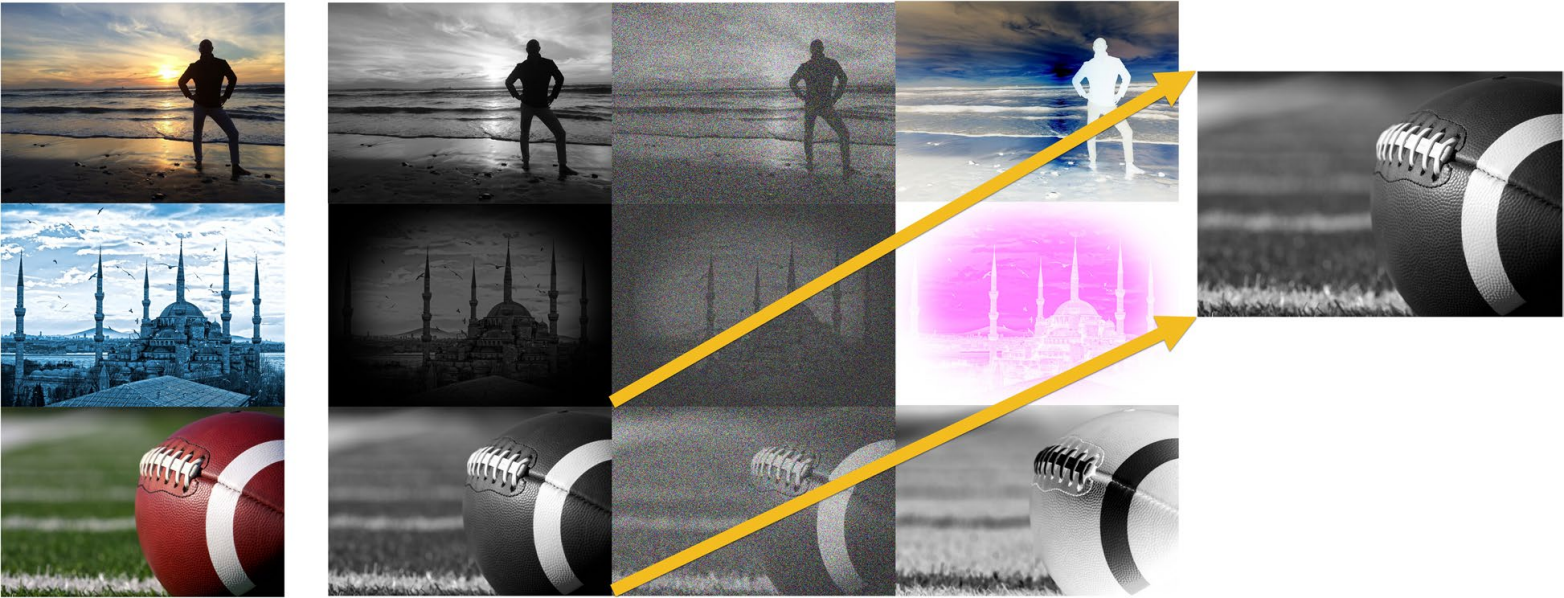
An example that clarifies the resultant deformations output through the ‘Preparation Network’

Regularly, with the existence of a size of M × M ‘Secret Image’ which was minor than the 'N × N' ‘Cover Image’, the ‘Preparation Network’ gradually raises the secret size image till reaching as the same size of the ‘Cover Image’; because of the ‘Secret Image’ distribution throughout the overall N × N pixels. ‘Preparation Networks’ convert color- based pixels into further usable characteristics, which defines deformations done mostly by the ‘Preparation Network’. This represents the function strength with all concealed images, regardless of their size, as represented in the Fig. 12 below, where:

- Left Part: Full-colored image.
- Center Part: ‘Preparation Network’ extracts data channels that represent the center network input.
- Right Part: Edge detectors scaling in.

The 'Preparation Network' alters the three-color streams; the second stream was activated for greater resonance frequencies.

The 'Hiding Network' represents the main network that imports both the ‘Cover Image’ and the 'Preparation Network' output and the resultant created part called the 'Container' image. The (N × N) represents the input size in pixels for this network, convolution ‘Cover Image’ RGB streams other than the modified ‘Secret Image’ streams with depth concatenation. More than thirty different network topologies were tested along with both 1. Variable hidden layer, 2. Convolution sizes. The output of five convolutions made up the optimum output out of 50 filters. Finally, the image receiver that represents the decoder uses the 'Reveal Network’, it gets only the 'Container Image' without neither both the secret nor the ‘Cover Image’; as the ‘Cover Image’ would be deleted by the decoder to expose the ‘Secret Image’. When both the ‘Preparation Network’ and ‘Cover Image’ were used together as inputs, the ‘Hiding Network’ constructs the Docker container. These channels were the depth combined:

1. RGB channels of the ‘Cover Image’.
2. The ‘Secret Image’ converted channels that were fed into this network.

**Fig. 13:**
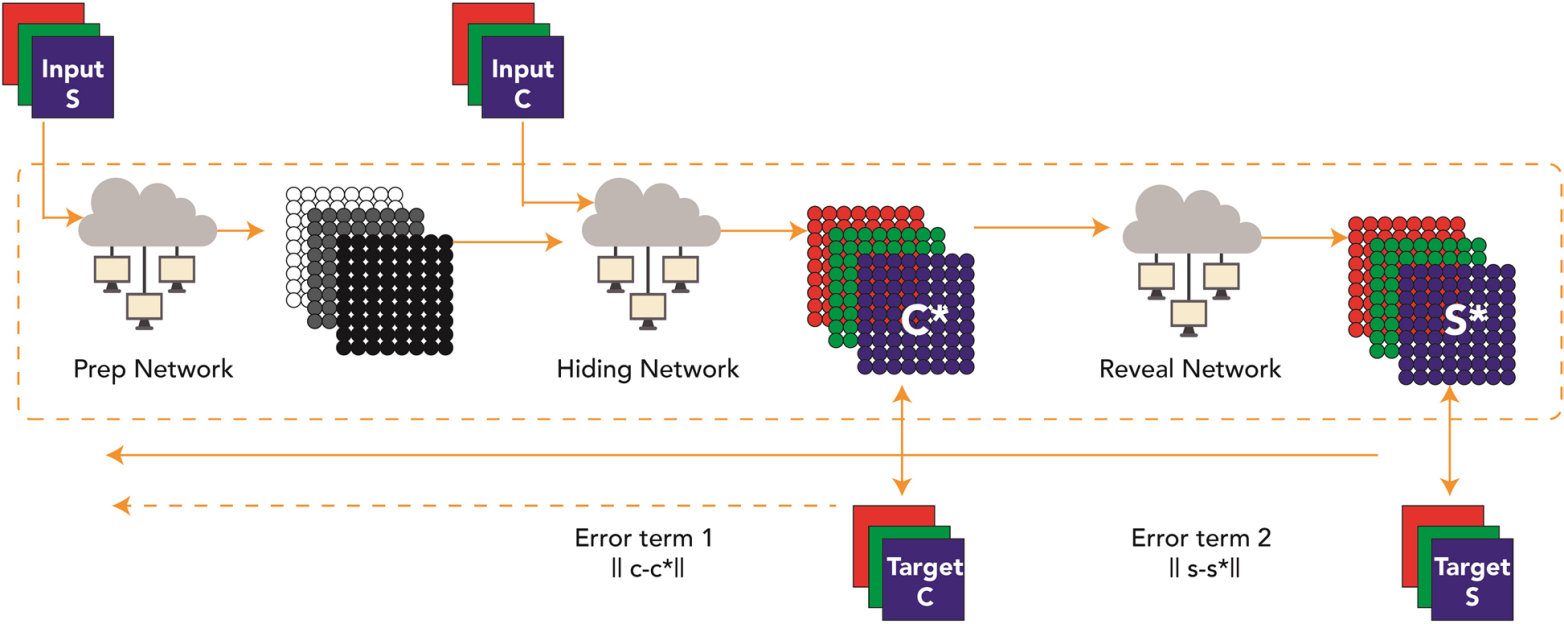
The three networks (Prep Network, Hiding Network and Reveal Network) were trained as one network, Error Term ||c - c^*^|| affected both (Prep Network) and (Hiding Network) while Error Term ||s – s^*^||

As stated previously, c = ‘cover image’, s = ‘secret image’ and β = ‘errors rate reformation’, both errors were measured along with every error impact. Specifically, the error part ||c − c^′^|| cannot be the same for the reveal network that imports the 'Cover Image' and exports the ‘Secret Image’. Contrariwise, all the error signals detected by the systems β ||s – s^′^|| for re-building the hidden image, through this way the process cycle that modeled earlier in the 'Preparation Network' is being controlled along with ‘Cover Image’ reconstruction. Figure 13 below shows the three networks during the training process for:

a- Preparation Network.
b- Hiding Network.
c- Reveal Network.

**Fig. 14:**
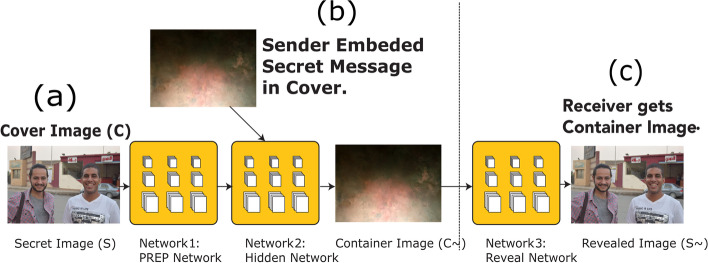
The proposed system in divided in three main parts. **a** Preparing the ‘Secret Image’, **b** Concealing the image through the cover image and **c** The reveal network usage for the hidden image exposure

The proposed framework designed in the Fig. 14 represents three trained phases in one network. Nevertheless, it was less difficult to divide them into three main networks to describe them easily. About 20 models of this network, including a different hidden layers’ number. In addition to convolutional sizes, were tested for our research; the optimum had five convolution layers using 35 filters. Eventually, the image transmitter utilizes the ‘Reveal Network’, which can be considered the decoder. Neither the cover nor the hidden images were sent to it. The hidden data was revealed after the decoder removes the ‘Cover Image’. During the network training phase, a small rate of noise volume was injected into the second network’s result (such as the created container image) to confirm that the hidden picture is not automatically encoded using LSBs. However, to prevent the hidden image recovery from being contained solely in the LSB, a reverse engineering was used towards the noise for reversing the LSB every now and then. Furthermore, a practical exploration to the network functions.

## Experiments

The implementation The implementation phase was accomplished by utilizing eight ASUS ROG Strix GL702VS with AMD Radeon RX 580 4 GB processor, RAM 32 GB DDR4 and a 512 GB SanDisk SSD hard disk with Windows 10 [92], BOINC 7.16.20 [93] and VMware 15.2 [94]. The optimal checkpoint rate was determined to be 15 per hour for a minimum of 2.52 GB of transmitted data from each ad-hoc client. If in a worst-case scenario, eight ad-hoc clients obtain a checkpoint from the transmitter, the transmitter is capable of transmitting 8.2 GB of data every hour. Consequently, a supposition would suggest that the ad-hoc cloud system has multiple hosts for each working visitor. Consequently, considering the execution increment rate of "ad-hoc guests" and "Cloud Jobs," the network failure rate increments for several checkpoints that are compiled in concurrently. The Cloud Job would be terminated if "Ad-hoc Client" assigns "Ad-hoc Guest" as inactive and sends feedback to "Ad-hoc Server". As indicated previously, in the event of a system guest failure, the ad hoc scheduler would select the optimal client for reintroducing the visitor into operation. While gathering requests for restoring the required checkpoint, the ad-hoc client would compile a series of successive events. According to the earlier 'Ad-hoc Client' evaluation of the assigned task in the overall ad-hoc system, the total operation will likely be completed within a minute.

**Fig. 15:**
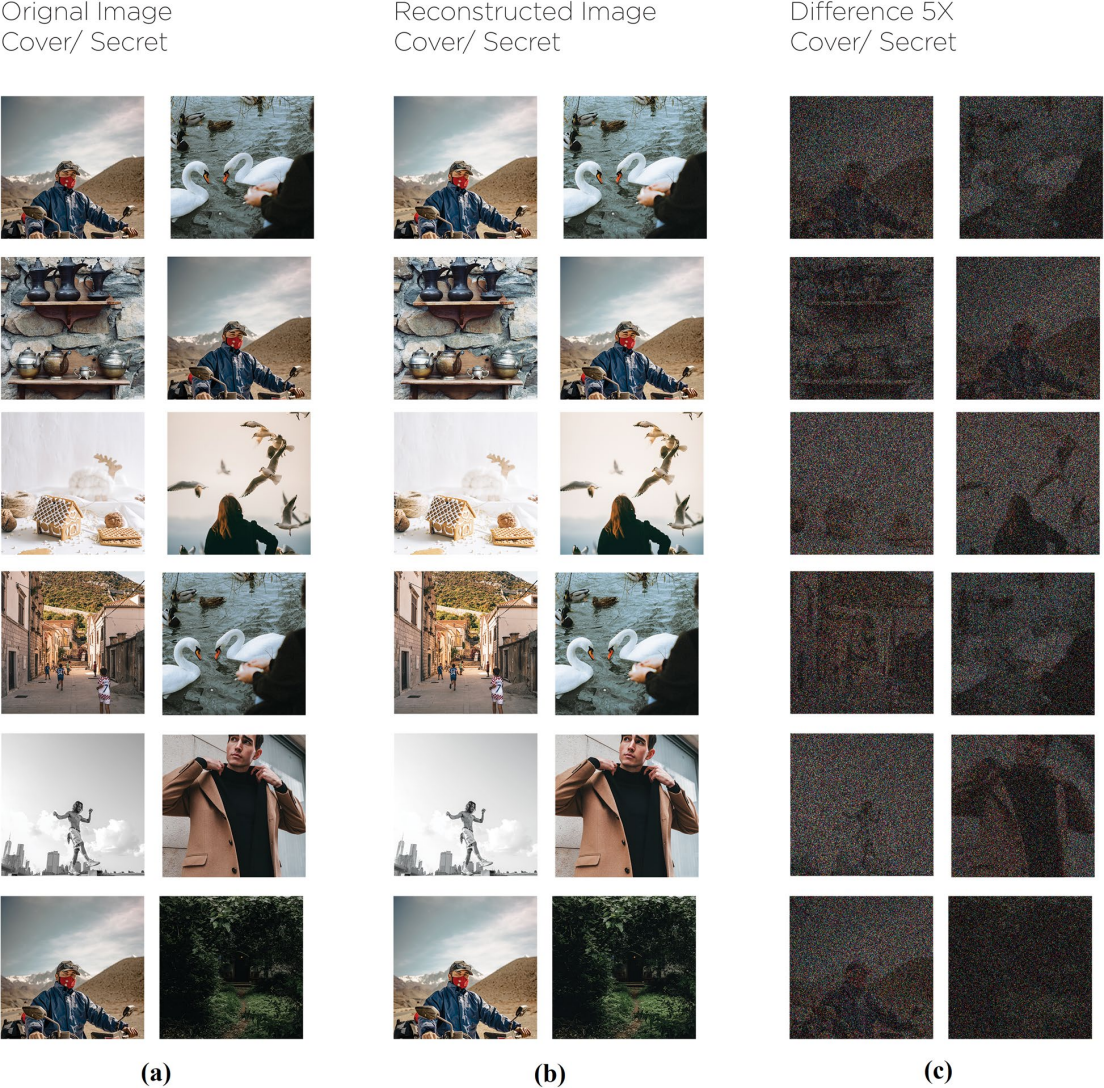
The results consist of three phases sets, **a** It includes both the main ‘cover image’ and *‘Secret Image’*, **b** Reconstructed Image and **c**) Secret/Cover error rate after improvement by 5x, the lowest error rate in the first row (2.8, 4.2) while the highest error rate in the last row (4.3, 7.8)

The primary focus was the testing phase of deep steganography operation performance on the 'Ad-hoc Server', to determine the server's ability to operate in the ad-hoc cloud system environment. AdaM [95] was used to construct the three networks, while ImageNet was the primary dataset utilized [96]. The testing portion of these networks comprised 1,000 photos from this dataset; to evaluate the final output of the 'Cover Image' encoding with no 'Secret Image' existence '= 0' across the same network, since it gives the optimal reconstruction of the 'Cover Image' failure through the same network. While it is possible to replace the picture measurements, there is an ongoing recovery process. Therefore, it can result in a total reduction of pixel variation. It is necessary to consider the error rate while constructing a container using LSB replacement [97]. Using an expected average, the persistent bits were reset to produce a fresh image in which the noise was fully concealed. Using this method to reconstruct the "Cover Image," the pixel intensity loss for each channel was 4.43 (scaling from 0.0 to 255). Using the median value for the deleted LSB bits would result in a maximum average reconstruction error of 3.81 bits. This inaccuracy of 3.0 or more was expected when the average value was used to fill in the LSB. Removing 4 bits from the encoding of a pixel reduces the number of intensities that may be represented by 16 times. By selecting the average value to replace the missing bits, the highest possible error is eight, while the average error is four, provided that bits are equally distributed. Consider using the average value for 'Cover Image' to avoid any confusion. Furthermore, the LSBs of the ‘Cover Image’ were stored where the ‘Secret Image’ MSBs were stored. Therefore, those bits must be used in this encoding scheme, and hence the larger error. The reason which led to the reconstruction cover image’s error being more than 3.81 is the usage of MSB from the ‘Secret Image’ instead of the LSB in the ‘Cover Image'. This results in a higher error rate than utilizing the LSBs average values, while both secret and cover samples were obtained within a similar range, as they were far superior to the ones in our current set-up, as can be illustrated from Fig. 15 below. If the approximate amount was utilized to change the LSB, the error rate of 4.0 was anticipated because 16x fewer intensity levels could be presented by eliminating 4 bits from the pixel's encoding. Considering equally distributed bits, using the arithmetic mean to replace lost bits results in a total deviation of 8 and a median failure of 4. However, the cover image’s LSBs were saved in the MSBs of the hidden image. For clarification, this encoding strategy requires the usage of those extra bits, which could lead to many mistakes. As a result, there was a need to demonstrate the drawbacks of this approach.

**Fig. 16:**
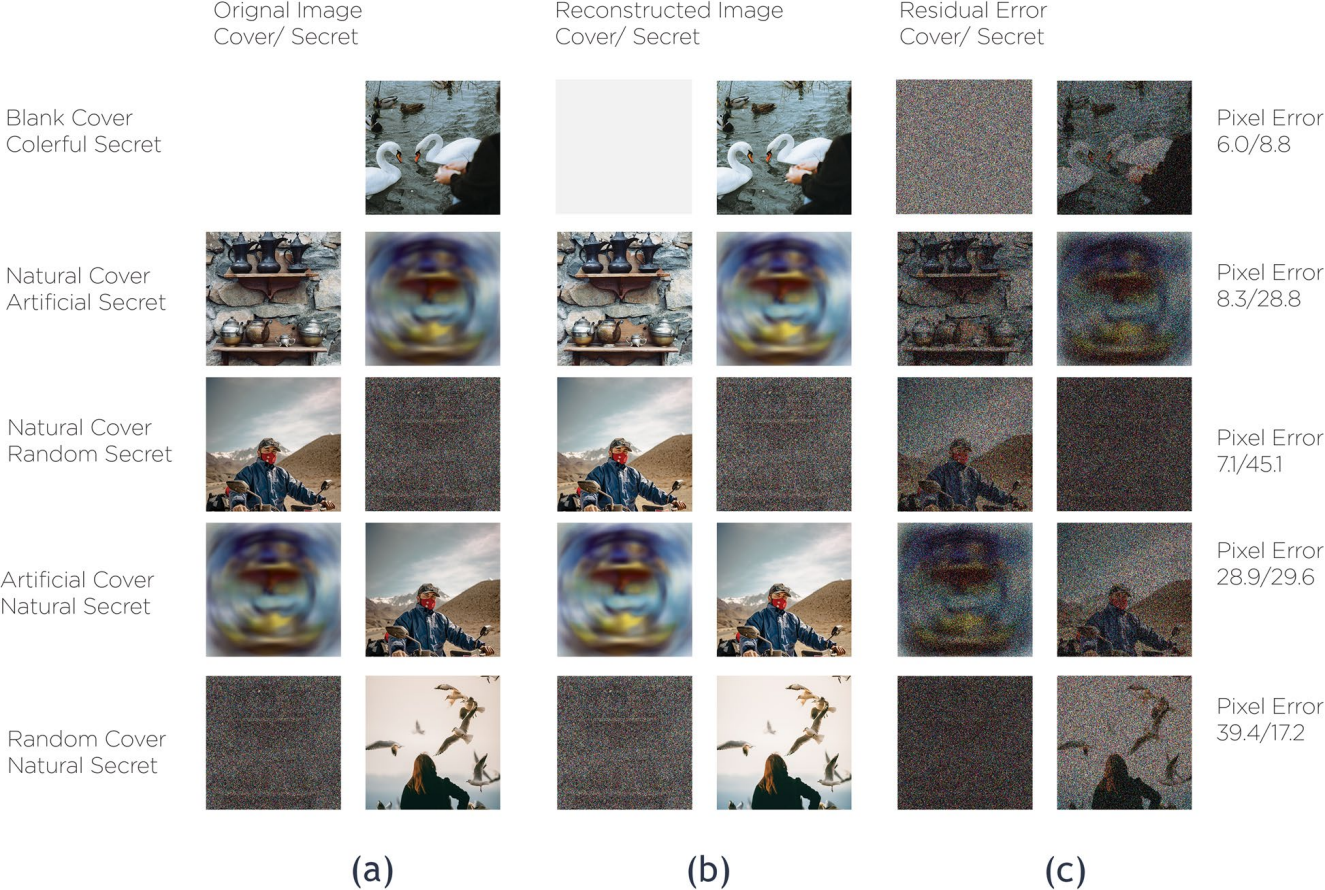
The images result in three doubled rows for ‘Cover Image’ and ‘Secret Image’ in three conditions: **a** Original Images, **b** Reconstructed Images, and **c** Residual Error, along with the pixel error rate on the right side

Several Steganographic approaches anticipate that an attacker does not have a high detectability rate to reveal the original ‘Cover Image’ (the encoded ‘Secret Image’ was not included) [98, 99]. However, in case the original image was discovered or whatever information might be gleaned regarding the ‘Secret Image’ in the case of the decoding network unavailability, As it is shown in Fig. 15, there was no difference between both the initial 'Cover–Container' and after it had been enhanced. It is essential to highlight that the training was conducted for networks using images from the (ImageNet) dataset, in which a wide range of images were covered. Nevertheless, it is important to analyze the impact when different types of features were employed. To illustrate this, five images were included in Fig. 16. Pure white images were utilized in the first row to monitor changes when a colorful ‘Secret Image’ was hidden. Using the ‘ImageNet’ dataset of images in training, this basic scenario was not really observed; the ‘Secret Image’ changes to consistent noise in the 2^nd^ and 3^rd^ rows. As from the observation, the retrieved ‘Secret Image’ was quite noisy, in spite of the container image ‘4^th^ column’ including only a small noise volume. Circles and consistent noise regarding the last two rows replaced the cover artwork. Recognized portions of both the "Cover Images" and the "Secret Images" could no longer be reconstructed due to considerable, but predicted, faults in the process.

**Fig. 17:**
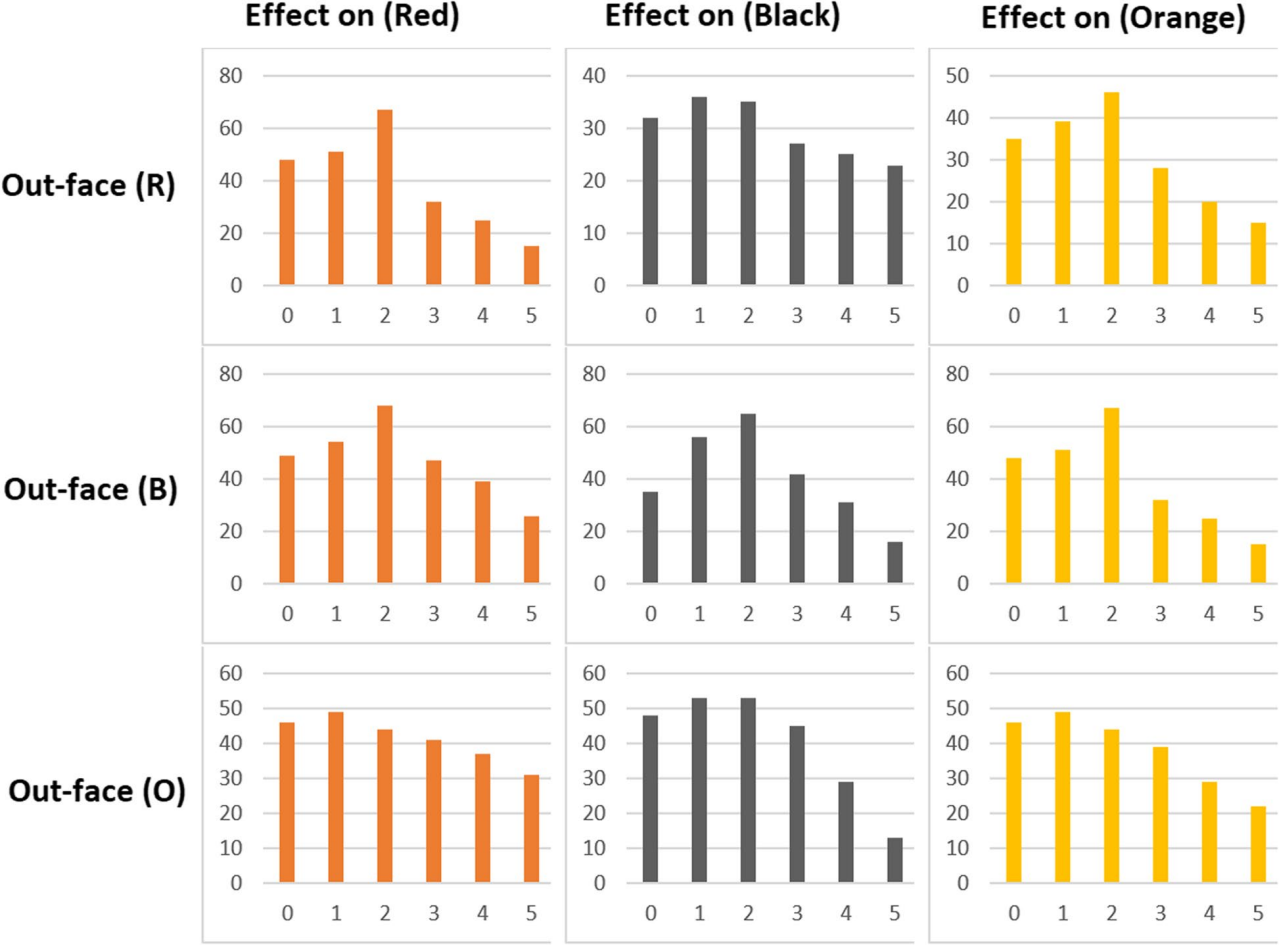
Changes in bits in the container image have the same effect on all the colors in the hidden picture that was retrieved

The left portion of Fig. 17 represents the control, as the statistics in the top-left corner represent the change in bit rate in only the container's red channel; similarly, the volume was correlated with the bit's significance; this is completely predictable, as the same can be said for both black 'B' and orange 'O' channels. Consequently, a similar bit flip within the container image affects all color characteristics of the retrieved "Secret Image. For instance, the reconstruction of the 'Secret Image' was affected by a single bit flip involving any color channel, which affects all color channels within the container image. In addition, the error disregarded the priority order of the bit positions. In addition, the data of the 'Secret Image' was dispersed across the color channels, which is why it had remained undetected until now. StegExpose did not find any encodings, but such a large volume of data could be easily discovered using other methods. Similar studies have demonstrated that deep neural networks can compete with and frequently outperform more established steganalysis techniques based on manually selected image characteristics. Numerous non-blind steganalysis techniques have been reported; as a result, they believe they can exclusively locate photographs that have been concealed using well-documented techniques. Therefore, it is easier to steganalyze but harder to conceal. Instead of examining the practical implications of this assumption, the author does not do so. From this, it can be concluded that any type of change to the bit position through a random channel in the 'Container Image' can have an effect on the creation of the 'Secret Image' via the overall color channels. In addition, there is no bit order positioning standard that the error can adhere to.

## Results discussion and performance analysis

**Fig. 18:**
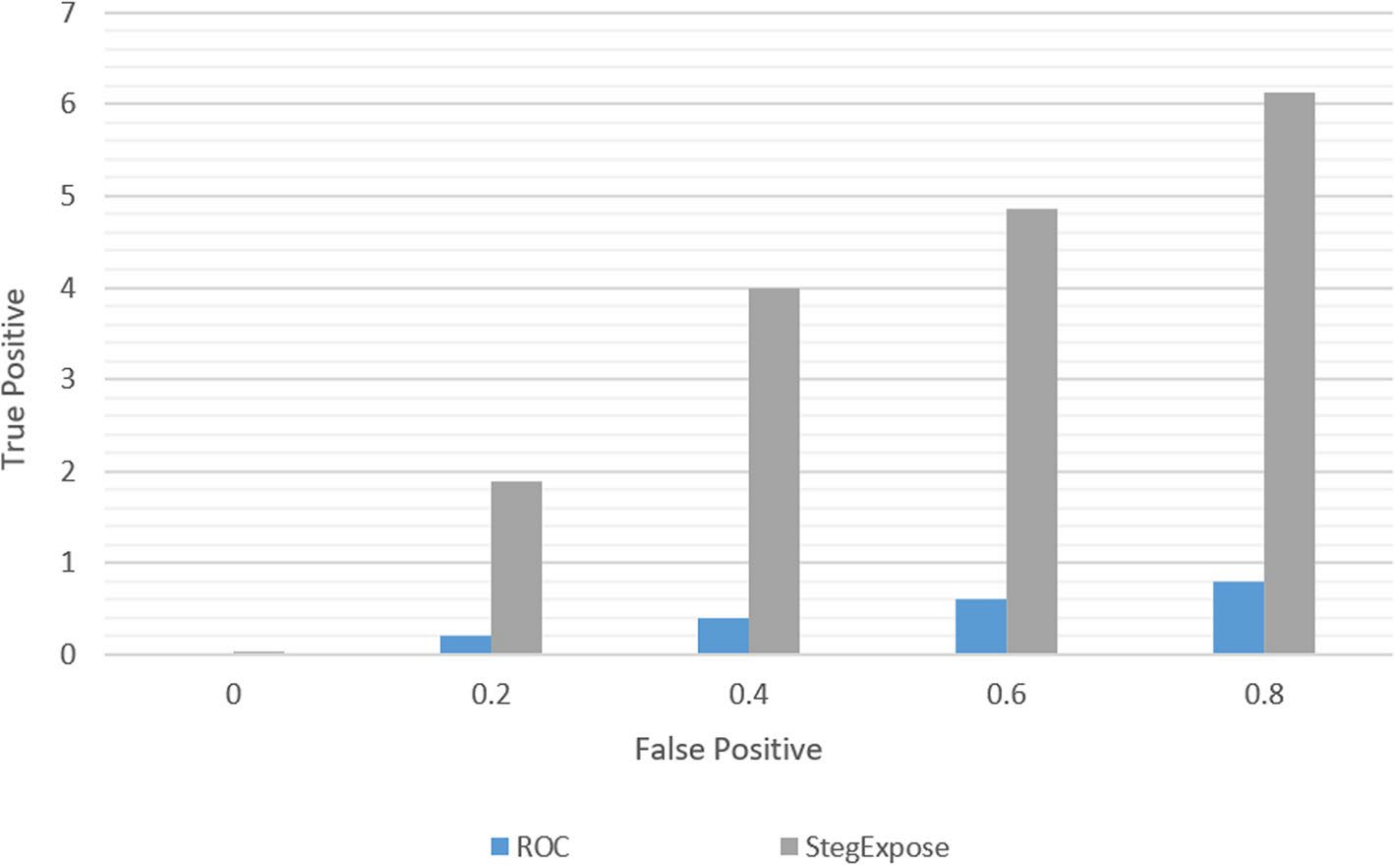
When using the proposed steganography analysis to identify hidden images, the rate comparison between (False Positive Rate) and (True Positive Rate)

The proposed model of ad-hoc cloud perception was evaluated in terms of security and reliability. The reliability was tested by using 9 nodes. In order to make an environment simulation with a reliable level, the ‘Nagios Network Analysis Tool’ was used for 14 days on 5 hosts. The resultant data of this monitoring operation was parsed and calculated for every host performance for an hour. The hour in which three hosts got the highest performance represents the optimum hour. Consequently, to test the security level, the focus was to enable the possibility of validation to encode large data volumes through an image using a restricted visually perceptible object that provides the ability to hide the data presence from being detected by a machine. At first, the ‘Secret Image’ data location should be identified to know in case the network was hiding the ‘Secret Image’ data in LSB for the ‘Cover Image’. Many existing tools were developed for the hidden data identification in the least significant bits. StegExpose was chosen to measure the detection rate of the hidden data in various tested samples. StegExpose is an open-source toolkit that is utilized in steganalysis. When the threshold was 0.15, the result was different all over a wider range. As represented in Fig. 18, the StegExpose numbers represent the receiver operating characteristics of the Report on Compliance (ROC) rate comparison between (False Positive Rate) and (True Positive Rate) during the embedded image detection operation through the proposed steganography approach. In previous studies, it was found that machine learning has the ability to compete or even outperform traditional ‘Steganalysis’ approaches, which rely on hand-picked image attributes. Even though different "Steganalysis" algorithms can find most of the hidden images using well-known hiding techniques, and even though the data access option for the cover image allocation is still available, this makes the work of "Steganalysis" much easier while making the job of hiding even harder.

### Virtual machine recovery process

**Fig. 19:**
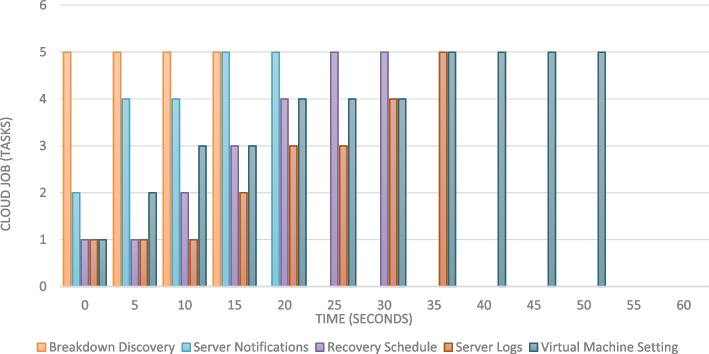
The recovery process overheads of (Ad-hoc Cloud System) through time in seconds

In the event that the ad-hoc server receives a notification, the 'Ad-hoc Cloud Hosts'-implemented checkpoints must be activated. The intended P2P strategy has conclusively determined the resultant overheads associated with periodic checkpoints as well as the potential for traffic management that were generated via a resource network. The VM recovery performance expenses must be considered when establishing a time limit for the Cloud Job. If an ad-hoc client determines that an ad-hoc user has stopped working, it notifies the (Ad-hoc Server) about the terminated (Cloud Job), the nearest optimal 'Ad-hoc Cloud Host' selected by the (Ad-hoc Scheduler) for the recovery process of the (Ad-hoc Cloud Guest), the decompression process for the checkpoint, and VM recording, and then completes the recovery. Figure 19 illustrates the processing times required to calculate the performance of a cloud-based operation over time. From the moment of the ‘Ad-hoc Client’ detection regarding any non-functioning ‘Ad-hoc Cloud Guest’ to the moment of the recovery of the "Ad-hoc Guest" on another "Ad-hoc Host". The overall operation takes around 60 seconds. The operation's overall time calculation was made through reliability measurement; the recovery process would averagely take about 35 seconds. In the event that any of the 'Ad-hoc Host' or 'Ad-hoc Server' goes down, the 'Ad-hoc Server' cannot detect failure. In the event of early detection of a failure, the recovery operation may take longer to complete, averaging about 120 seconds on average. Furthermore, an assumption was made that availability for one ‘Ad-hoc Host' should minimally exist to schedule the recovery through the ad-hoc scheduler. On the contrary, the time it takes the ‘Ad-hoc Host’ to be accessible could make the complete time for system recovery longer as well; it should be mandatory for the ‘Ad-hoc Client', which was chosen by the ‘Ad-hoc Host', to make a checkpoint recovery. Lastly, in the event of the recovery operation's success, the client side would notify the "Ad-hoc Server".

### Cloud system performance evaluation

**Fig. 20:**
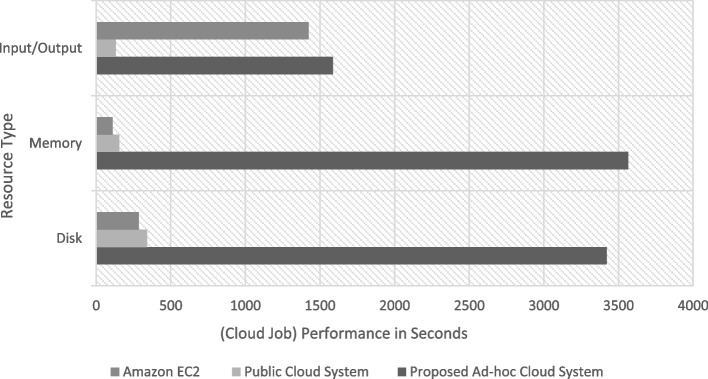
A comparison of the proposed (Ad-hoc Cloud System) and (Amazon EC2) performance metrics (Input/output rate, Memory utilization, and Disk space)

The execution level comparison of 'Cloud Jobs' between the 'Ad-hoc Cloud System' in the proposed study and a general host was performed on the Amazon platform. Specifically, the evaluation includes execution time in addition to the checkpoint and VM recovery operations. The total operation time was determined from the time the job was submitted until its completion. The checkpoint configuration has been set to 50/hour in an effort to incorporate the VM recovery time. The transaction has already taken approximately 35.8 seconds to complete. Memory, I/O, and Disk resource executions were performed on the Ad-hoc Cloud System prototype. Figure 20 depicts the results of a comparison of resource use over time between the suggested ad-hoc cloud prototype, a typical public cloud, and Amazon EC2. As anticipated, there was a significant variation in the overall (Cloud Job) execution time between the proposed ad hoc cloud prototype, the public cloud, and Amazon EC2. The variance in processor timing was caused by hardware-based virtualization, which results in a lower throughput than Amazon EC2. However, the overall execution time can be reduced if no migrations occur during the procedure. On the other hand, it was projected that the overall amount of time would increase by 15% to 25% for each migration procedure that occurred within the system.

### The (ad-hoc cloud server) performance

**Fig. 21:**
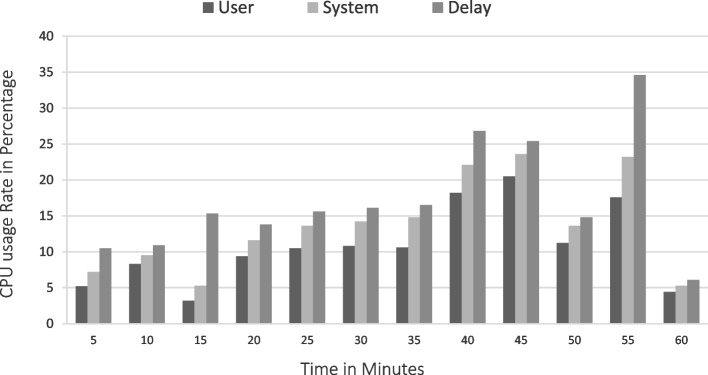
CPU usage rate in percentage measurement in the (Ad-hoc Cloud Server) within 60 min

**Fig. 22:**
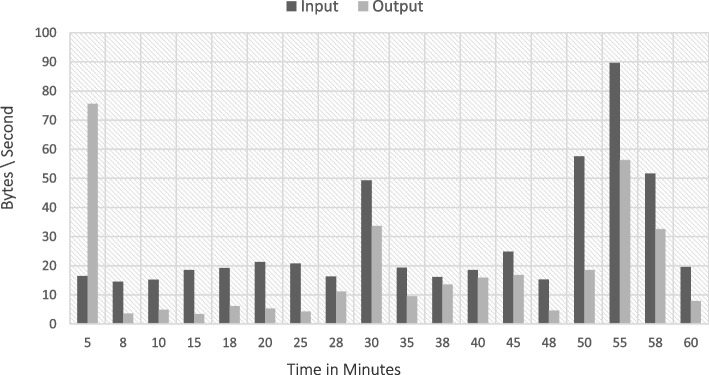
The data input/output in bytes per second for the ‘Ad-hoc Cloud Server’ through 60 min

Subsequently, the focus would be on evaluating the performance of the ‘Ad-hoc Server’ that was implemented in our experiment; to measure the operation level the server can reach within the ad-hoc Cloud prototype simulation. The ‘Ad-hoc Cloud Server’ was observed through the (Command and Control Message Specification) through the CPU within one hour. As illustrated in Fig. 21, the ‘Ad-hoc Cloud Server’ has used its two main processes within the 12 minutes of experiment, it was noticed a huge increment in CPU usage due to ‘BOINC daemon’ that was utilized in both ‘Ad- hoc Server’ and ‘Ad-hoc Clients’; as the utilized memory were 3.56 GB out of available 4.0 GB. The limitation in this part is not in the CPU usage level, but due to the work-units that were hosted on multiple VMs in the ‘Ad-hoc Cloud Host’ was the main reason for the high CPU usage, which can represent an over CPU capacity usage on large scale networks, as illustrated in Fig. 22.

**Fig. 23:**
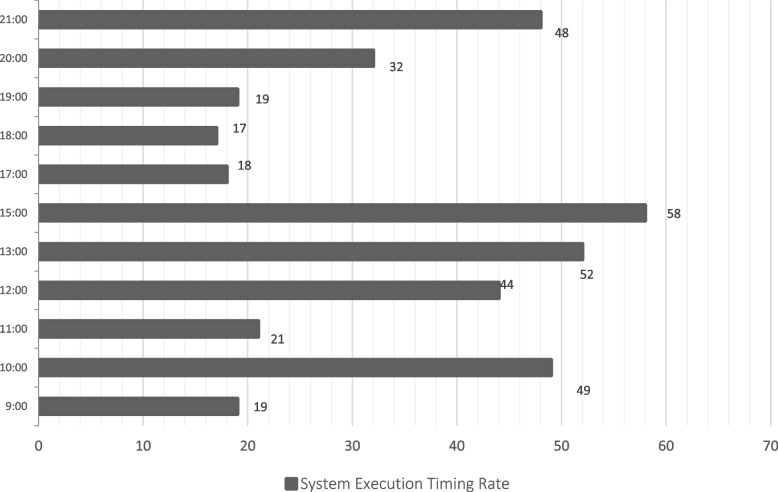
The time that has been taken by the (Ad-hoc Cloud System) to execute referred as the ‘Execution Time) in seconds throughout the daytime in hours

As shown in Fig. 23, there can be a major difference regarding the software execution time. In this study, the evaluation of both the lowest (41 minutes) and the highest execution time (82 minutes) has been done for 12 hours continuously. This difference in time was still not acceptable as it could result in high costs for the user for each usage hour. As an example, if the user tried to execute software at 8:00 am, it might take 82 minutes at most to finish the task, which is still considered a limitation; for each additional minute, the user might be charged with a higher cost.

### Data utilization charging method

**Fig. 24:**
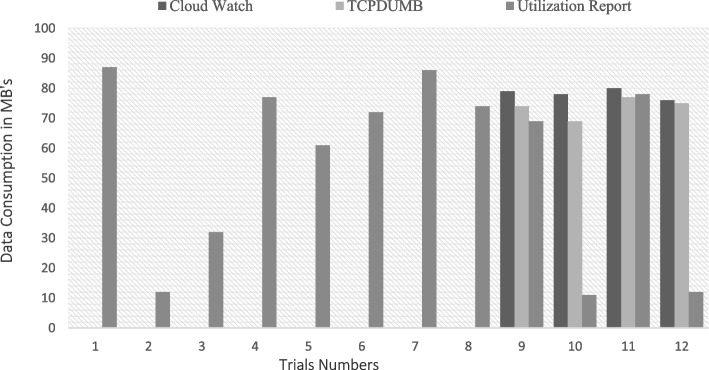
Measuring the data transmission between the implemented (Ad-hoc Cloud System) through the utilization report and (Amazon EC2) platform through the Cloud Watch

The service (Pay as You Go) that was being delivered by Amazon EC2 was charging its users for using both cloud resources and data packages in GB for every passing hour [100]. The charging rates for both ‘Storage’ and ‘Instance’ were low when compared to the ‘Data Transfer’ rate charging, as there were various complex matrices involved during the transfer (i.e. packet size, transferred packets numbers, and data type) for instances and the user. The test here is to evaluate Amazon EC2’s designed transfer approach for controlling the cloud users during data transfer. The evaluation was carried out on a CentOS 7.2 i386 server [101], with various types of data transferred from numerous cloud users, and a comparison made between the actual data transferred and the transferred data measured by Amazon EC2.As an example, an installation for a random application, the packages for the application were installed on both the local machine of our implementation and the CentOS 7.2 on (Amazon EC2), as illustrated in Fig. 24. “Cloud Watch” is a packet analyzer tool, which was used to measure the volume of the transferred data every hour, while the utilization report from our local machine was used from the other side.

### Deep steganography performance results

**Fig. 25:**
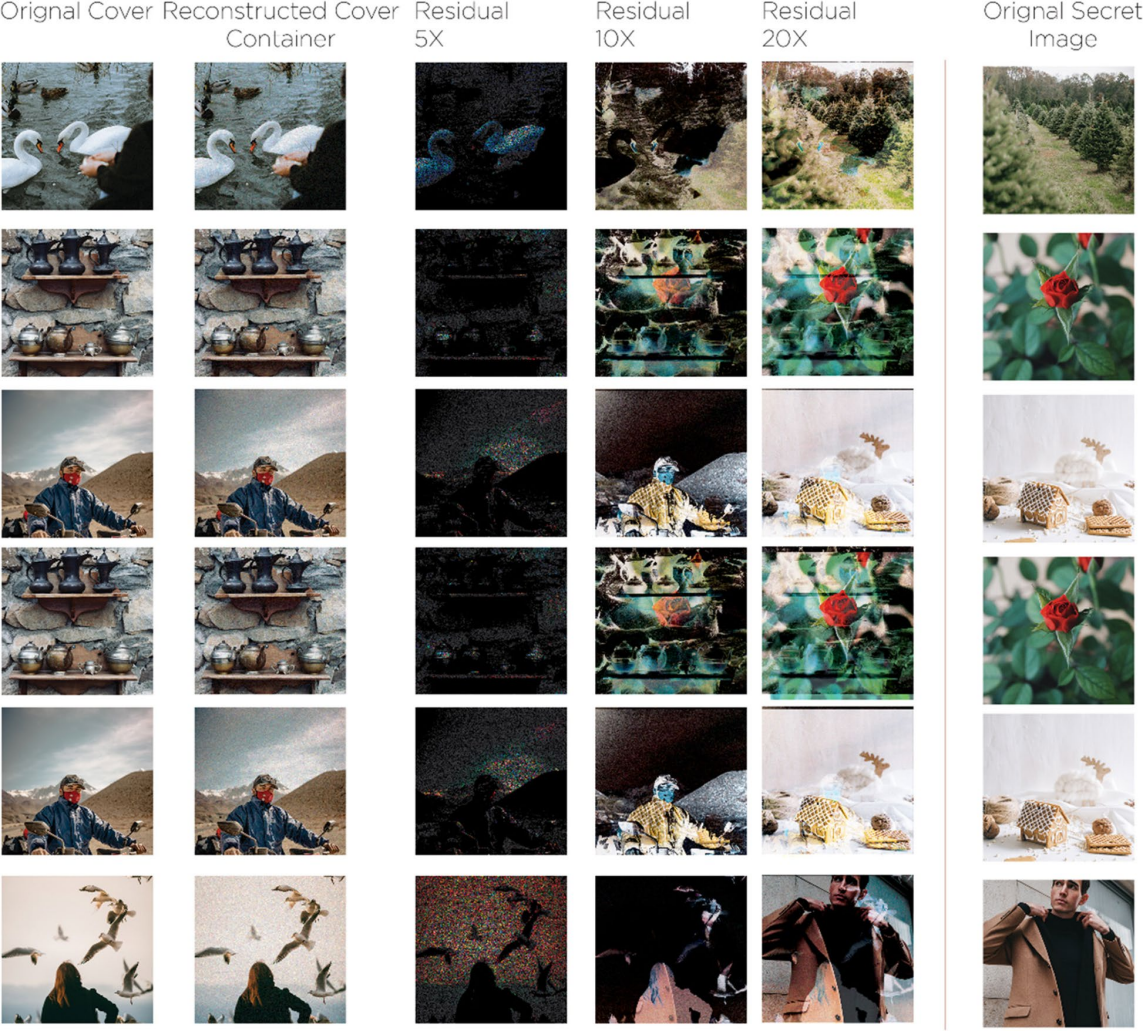
A description of the residual image computation that can be achieved in case the container image substation during the original image leakage, as it shown in column 3, 4 and 5 then enhancement output can be seen in 3rd, 4th and 5th rows with enhancement rate of 5*x*, 10*x* and 15*x* of residual image

As shown in Fig. 25, the enhancement level of the remaining image can be increased by five, ten, or twenty times.

- First Row: The residue matching high rate regarding the ‘Cover Image’ (at 20x).
- Second Row: It contains a combination result of both types of images (cover - hidden).
- Third Row: ‘Secret Image’ aspects were exposed.

The comparison can only be done with an original image that has not been changed. One standard way to hide a “secret image” once it has been placed in a “cover image” is to use cryptographic encodings. As an extra choice, we show how to change the network’s error function. It was the relationship between the cover image’s residual and the secret image’s corr (Rc, S), where Rc = ||C C′|| and S denotes the ‘Secret Image’, that caused the most concern. Various several distinct definitions of this term were evaluated. In the results given in Fig. 25, the scale was assessed to be (0.15 x Channels x Number of Pixels). Even with 20x of residual image enhancement, various ‘Secret Image’ characteristics were eliminated; this was accomplished by minimizing the residual correlation only with encrypted images; because of their strength and sturdiness, the reconstructions’ integrity has degraded in a few random places; the objective of this section was to demonstrate that a significant data volume could be encoded via a single image while still leaving several discernible artifacts. Despite this, no effort has been made to conceal the existence of this data from machine detection. Since up to the majority of a document’s contents are hidden messages, numerous measures can be used to make it more difficult to decode. In order for the network to conceal the existence of the secret image, it was necessary to determine the location of the hidden image’s data. Inconsequential cover image fragments were insufficient to disguise the secret image’s existence. The LSBs may include data that might be revealed using a variety of methods. StegExpose, a free toolbox for steganography, was used to see if the hidden images could be found.

### Testing the deep steganography performance towards multiple attacks

**Fig. 26:**
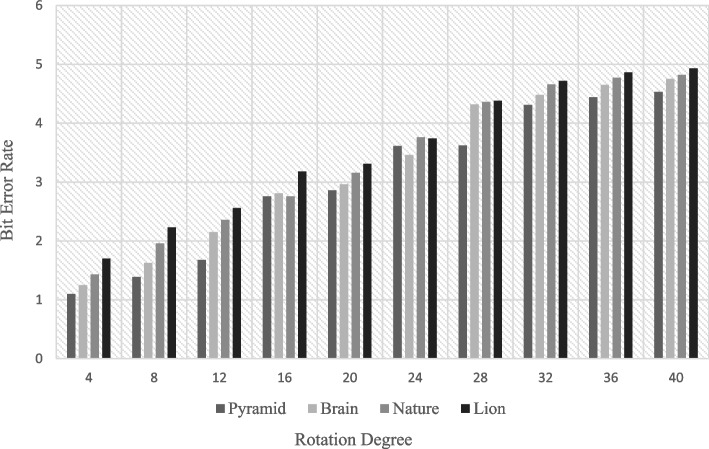
The (Bit Error Rate) results towards different image’s rotation degrees

In case for an accessibility to un-authorized users, the (Geometrica Attack) has a high rate of resistance to images with watermarks, the danger of these attacks is that they have the ability to alter both of the image’s ‘data’ and ‘features’ as the output of a deformed version. In this study, both (Rotation Attacks) and (Cropping Attacks) were considered. Harmonic transformation was mainly utilized for image rotation, the output of pixels’ displacement, whether in clockwise motion or anti-clockwise motion, the (Rotation Attack) can cause an image deformation output along with edge pixels that have an arrange of triangle model, the resultant effects of the (Rotation Attacks) on watermarked images can be simulated based on the image’s rotation degree as illustrated in Fig. 26.

**Fig. 27:**
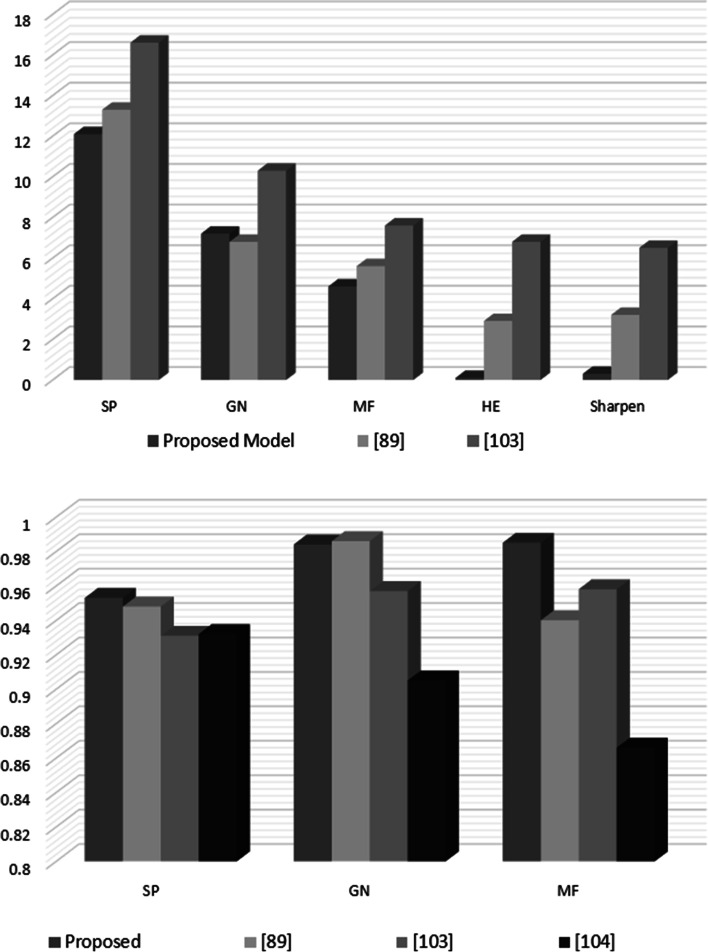
The evaluation of ‘Pyramid’ image, **a** comparing the (Bit Error Rate) for gray scaling, **b** comparing the (Normalized Cross Correlation) for color scaling when it is compared to the studies in [89, 103, 104]

From the resultant output, it possible to state that the case in which the watermarks obtainability relies on the rotation range. As an instance, the implemented technique has a BER lower than 10% when the rotation degree is 50. In Fig. 27, ‘Pyramid’ image can compare the (Normalized Cross Correlation) proposed approach comparison to illustrate the (Rotation Attack) outcome on each angle. The proposed methodology has the ability of attaining high performance towards the (Rotation Attacks); that’s due to arbitrary block and selection parameters, the random selection role ensures that in case any type of modifications is made regarding the rotation of the watermarked images. In Table 3 below, it presents the output rotation results based on both (Image’s Attacks) and (Watermark Extraction), in case of the metrics results were presented genuinely, there is a 15^o^ rotation for multiple gray scale images. (Cropping Attack) is a type of attacks in-which it often replaces image portions (i.e. Square, Circle or Rectangle) with white/dark pixels [102]. The difference in the cropped image phase that it can start from 1% at least until 100% at most. For a simulation testing, some random crops were made to the ‘Pyramid’ image at various parts; this resulted in a high extraction image quality from the cropped images.

**Table 3:**
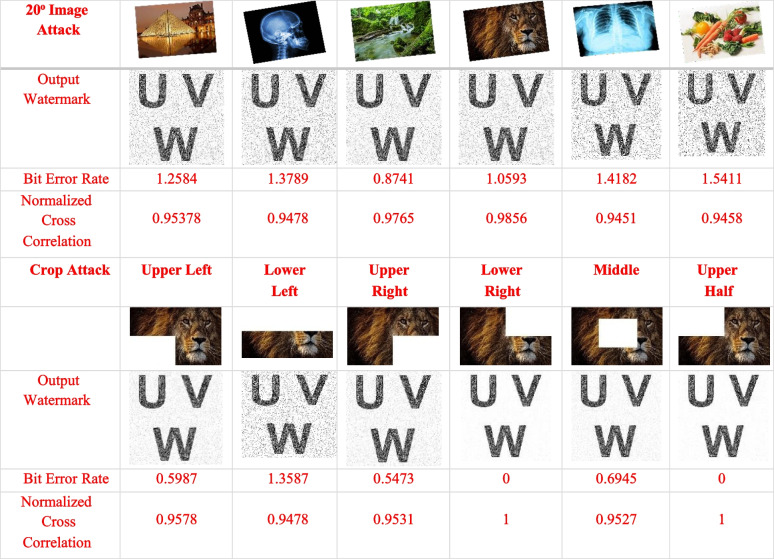
The output watermarks of both a) rotated images with 20^o^.b) various cropped lion images

Regarding rotation, as previously stated, the performance best case of the implemented technique concerning cropping attacks for embedding is caused by both (Arbitrary Block) and (Selection Parameters). ‘Pyramid Image’ was used to test the implemented technique efficiency. During the transmission phase of the watermarked images, many multiplicative noises would be collected though all over the image; to evaluate the implemented approach’s performance. Several watermarked images were tested using multiple noise attacks with multiple densities of both (Output Watermarks) and (NCC - BER), the images were evaluated under case (Variance = 0.005, Mean = 0, Noise Density = 0.05).

There is a high resistance level towards noise attacks in this study along with high robustness level towards (Histogram Attacks). Some limits in the performance can be shown with lower results (i.e. Gaussian Noise). Correspondingly, as it can be illustrated in Fig. 27, the resultant output form the proposed approach when evaluated towards several types of attacks were put into comparison with [89, 103, 104].

From other different steganography methods, the proposed steganography approach has provided a high output results, which has the ability to hide data\image through the deep learning usage [105], which was designed from three main networks (Preparation Network, Hidden Network and Reveal Network), the extraction possibilities by various method is low. Consequently, there is an elasticity to change the data\image concealing operation, which therefore lower the extraction possibilities for the hidden data\image. It is important to mention that the extraction threat level can be higher if the attack came within the same ad-hoc cloud node [106]. Generally, it is approved that the designed hiding data\information approach provide a high positive rate of data transmission security through the (Ad-hoc Cloud System).

### Study limitations

If one of the two images (Cover Image / Secret Image) was additionally provided, the network might be trained to retrieve all these constituent elements in every one of the repeated versions of container images made by the target machine. There can be an appliance for a simplicity restriction and perhaps other image deconstruction or blind sources detachment approaches to see if it can limit the attacker’s options if they do not have accessibility for the "training" data. Getting a small amount of training data would be helpful for defining variables and priors to many of these approaches. However, the following steps should be followed to enhance the system robustness:

- After concealing the hidden image, the pixels should be permuted (in-place) in one of M agreed on techniques; system subsequently hides the permuted-secret-image as well as key (an index into M).
- The lack of structural configuration throughout the residuals makes recovery significantly more challenging, unless the original image was accessible.
- Permutation keys were required for this method to work properly (though this can be sent reliably in only a few bytes).
- There were various complications for transmitting a concatenated ‘Secret Image’, which increases the probability of reconstruction errors across the system.

## Study conclusions

This paper has discussed data security in ad-hoc cloud systems through the usage of enhanced steganography approach with the usage of deep learning. The ‘Ad-hoc Cloud System’ platform idea along with its deployment approach were proposed in this work. An end- user's hardware was leveraged to launch a cloud feature on an irregular basis. V-BOINC is an open-source tool, which allows developers to bypass application-level security checkpoints by solely using the V-BOINC VMs. The ad-hoc cloud approach can help enhance network performance along with usage while lowering costs. Secondly, this research expands the study of steganography, then the utilization of pertinent data in images through the usage of deep learning. Prior attempts to employ machine-learning models to supplement or replace an image-hiding scheme ratio have failed. A fully trainable deep learning system was created consists of three networks which seamlessly inserts a color image into another one. The scheme would be designed to insert data or another image. Implementing a steganography technique through the usage of a deep learning approach, the message must be hidden from statistical analysis. It would require an additional training target and possibly embedding tiny images beneath many ‘Cover Images’. No image loss sources would be re-used with the suggested extracted features. The re-training phase of the already trained networks is a required part of this strategy. Since hidden systems would no longer take advantage of the local architecture in the concealed image, the rest of the system should be retained. Using steganography and deep learning techniques for hiding additional data in photographs in the proposed solution has never been more accessible. More than one previously proposed technique has attempted to use neural networks including a replacement for some tiny component of an image-hiding network. It has been proved where a completely resilient system that produces visually good performance in the placement of a full-size, color image further into a picture has a possibility to be created. This was discussed in terms of graphics, but the same system can be trained to embed text or image as well, the project's potential for growth is limitless, both in the short and long terms, these three were mentioned based on the priority level.

- In order to create a holistic steganographic scheme, the methodology of concealing the message existence in the statistical analyzer must be addressed, as it most likely demands a new objective in training, in addition to having a small image encoding technique in a larger ‘Cover Image’.
- The proposed embeddings that were addressed in this study were not planned to be used with image loss files, if the lossy encodings, (i.e., jpeg, bmp, or png) were needed, then there was a possibility to work directly with the DCT coefficients rather than the spatial field.
- The SSE error unit was utilized for the networks’ training. However, error units associated with SSIM may be easily removed.

## Future works

The proposed study has provided high positive results; there are huge room for improvements for future studies regarding the previously mentioned limitations in section 6. The reliability issue represents a real critical point to be discussed as a separate study; because resource load capabilities were not included regarding the measuring the reliability features [107]. For instance, the ad-hoc cloud system reliability computations were saved within the VM Service project DB; these computations provide the needed estimations for the 'Ad-hoc Cloud Host' behavior. However, there is a possibility that the reliability computations could be modified for the host reliability measurement (i.e., the host's weekly/monthly patterns might be utilized for allocating a ‘Cloud Job’); it is left for future studies to investigate new approaches for solving such issues [108]. Further future studies could be conducted to see if the methods discussed in the presented work can be modified to test the affection level in a case of a cyberattack incident within a cloud environment. The hidden image's presence (but not its specific composition) could be accurately detected, in comparison to the data of the ‘Cover Image’ information, this became significantly beyond a state-of-art framework [109]. Unless the cover image's residual has a low enough measure of correlation to the concealed image, it becomes harder to decipher its elements [110]. Hence, future studies could be focusing into providing an effective two-factor encryption mechanism within a normal public cloud as a start.

## Data Availability

The data that support the findings of this study are available from the corresponding author Ahmed A. Mawgoud, upon reasonable request.
